# Person Re-identification in Identity Regression Space

**DOI:** 10.1007/s11263-018-1105-3

**Published:** 2018-07-27

**Authors:** Hanxiao Wang, Xiatian Zhu, Shaogang Gong, Tao Xiang

**Affiliations:** 1 Electrical and Computer Engineering Department, Boston University, Boston, MA 02215 USA; 2 School of Electronic Engineering and Computer Science, Queen Mary University of London, London, E1 4NS UK; 3 Present Address: Vision Semantics Limited, London, E1 4NS UK

**Keywords:** Person re-identification, Feature embedding space, Regression, Incremental learning, Active learning

## Abstract

Most existing person re-identification (re-id) methods are unsuitable for real-world deployment due to two reasons: *Unscalability to large population size*, and *Inadaptability over time*. In this work, we present a unified solution to address both problems. Specifically, we propose to construct an identity regression space (IRS) based on embedding different training person identities (classes) and formulate re-id as a regression problem solved by identity regression in the IRS. The IRS approach is characterised by a closed-form solution with high learning efficiency and an inherent incremental learning capability with human-in-the-loop. Extensive experiments on four benchmarking datasets (VIPeR, CUHK01, CUHK03 and Market-1501) show that the IRS model not only outperforms state-of-the-art re-id methods, but also is more scalable to large re-id population size by rapidly updating model and actively selecting informative samples with reduced human labelling effort.

## Introduction

**Fig. 1:**
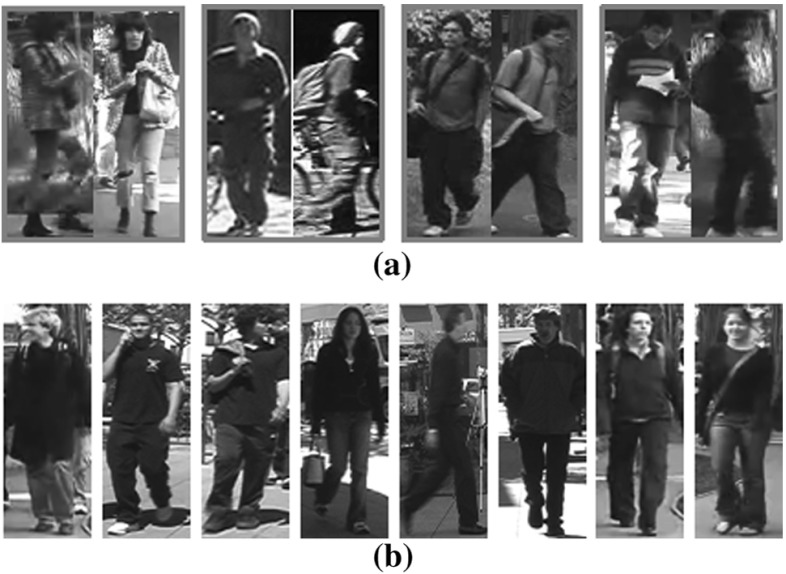
Illustration of person re-identification challenges. **a** Significant person appearance change across camera views. **b** High visual similarity among different people

Person re-identification (re-id) aims to match identity classes of person images captured under non-overlapping camera views (Gong et al. 2014). It is inherently challenging due to significant cross-view appearance changes (Fig. 1a) and high visual similarity among different people (Fig. 1b). Most existing re-id methods focus on designing identity discriminative features and matching models for reducing intra-person appearance disparity whilst increasing inter-person appearance individuality. This is often formulated as a supervised learning problem through classification (Koestinger et al. 2012; Liao et al. 2015), pairwise verification (Li et al. 2014; Shi et al. 2016), triplet ranking (Zheng et al. 2013; Wang et al. 2016d), or a combination thereof (Wang et al. 2016a). While achieving ever-increasing re-id performance on benchmarking datasets (Zheng et al. 2016; Karanam et al. 2016), these methods are restricted in scaling up to real-world deployments due to two fundamental limitations:

**(I)** *Small Sample Size* The labelled training population is often small (e.g. hundreds of persons each with a few images) and much smaller (e.g. $<\frac{1}{10}$) than typical feature dimensions. This is because collecting cross-view matched image pairs from different locations is not only tedious but also difficult. The lack of training samples is known as the small sample size (SSS) problem (Chen et al. 2000), which may cause singular intra-class and poor inter-class scatter matrices. Given that metric learning re-id methods aim to minimise the within-class (intra-person) variance whilst maximising the inter-class (inter-person) variance, the SSS problem is therefore likely to make the solutions suboptimal.

**(II)** *Inadaptability* Existing re-id methods often adopt off-line batch-wise model learning with the need for sufficiently large sized training data collected via a time consuming manual labelling process. This *first-labelling-then-training* scheme is not scalable to real-world applications that require deployments at many previously unseen surveillance locations with little or no labelled data in advance. Also, real-world label collection is more incremental, i.e. additional label data are sequentially available for model update over time. It is hence desirable for a re-id model to grow and adapt continuously to progressively available up-to-date labelled data. Existing re-id methods can only afford model re-training from scratch, causing both high computational cost and response latency to a user. They are thus unsuitable for human-in-the-loop model adaptation.

In this work, we solve the two issues by formulating person re-id as a *regression* problem (Hoerl and Kennard 1970). Unlike existing methods designed to learn *collectively* from all the training identities a *generic* feature embedding space optimised for classification, verification or ranking, we propose to construct an *individually* semantic feature embedding space for identity regression optimised on *each training identity*, referred to as an *identity regression space* (IRS) defined by all training identity classes. Each dimension of IRS corresponds to a specific training person class, i.e. all training images of the same identity class are represented by a single unit vector lying in one unique dimension (axis). Our modelling objective is therefore to train a regression model that maps (embeds) the original image feature space to this identity regression space.

We formulate a re-id incremental learning framework with three fundamental advantages: *First*, it allows quicker re-id system deployment after learning from only a small amount of labelled data. *Second*, the learned re-id model facilitates the subsequent labelling tasks by providing human a ranking order of unlabelled samples with the labelling targets (i.e. true matches) in top ranks at high likelihoods. This reduces manual search time and effort as compared to the conventional exhaustive eye-balling of unstructured person images. *Third*, the re-id model progressively improves from new labelled data to further facilitate future labelling. This interactive effect is cumulative in a loop: More frequently the model updates, more benefit we obtain in both reducing labelling effort and increasing model deployment readiness.

Our **contributions** are threefolds: (**1**) We propose the concept of an *identity regression space* (IRS) by formulating re-id as a regression problem for tackling the inherent small sample size (SSS) challenge. This is in contrast to existing methods relying on classification, verification, or ranking learning spaces which are subject to the SSS problem. The IRS model is featured by an efficient closed-form feature embedding solution without the need for solving an expensive eigen-system and alternative optimisation. (**2**) We introduce an incremental learning algorithm for efficient on-line IRS model update. This facilitates rapidly updating a IRS re-id model from piecewise new data *only*, for progressively accommodating update-to-date labelled data and viewing condition dynamics, hence avoiding less efficient model re-training from scratch. (**3**) We develop an active learning algorithm for more cost-effective IRS model update with human-in-the-loop, an under-studied aspect in existing re-id methods. Extensive experiments on four popular datasets VIPeR (Gray et al. 2007), CUHK01 (Li et al. 2012), CUHK03 (Li et al. 2014) and Market-1501 (Zheng et al. 2015) show the superiority and advantages of the proposed IRS model over a wide range of state-of-the-art person re-id models.

## Related Work

**Person Re-ID** Existing person re-id studies focus on two main areas: feature representation and matching model. In the literature, a number of hand-crafted image descriptors have been designed for achieving general non-learning based view-invariant re-id features (Farenzena et al. 2010; Zhao et al. 2013; Wang et al. 2014a; Ma et al. 2012; Yang et al. 2014; Matsukawa et al. 2016). However, these representations alone are often insufficient to accurately capture complex appearance variations across cameras. A common solution is supervised learning of a discriminative feature embedding, subject to classification, pairwise or triplet learning constraints (Liao and Li 2015; Wang et al. 2014b, 2016a).

Our work belongs to the supervised learning based approach but with a few unique advantages. *First*, our IRS is designed with each dimension having discriminative semantics, rather than learning to optimise. We uniquely train a regression mapping from the raw feature space to the interpretable IRS with a close-formed optimisation solution (Hoerl and Kennard 1970; Hastie et al. 2005) more efficient than solving eigen-problems (Liao et al. 2015; Zhang et al. 2016a) and iterative optimisation (Zheng et al. 2013; Liao and Li 2015). The IRS addresses the SSS problem in a similar spirit of the NFST re-id model (Chen et al. 2000; Yu and Yang 2001; Zhang et al. 2016a) by projecting same-identity images into a single point. Importantly, our model uniquely confirms to a well-designed embedding space rather than relying on intra-person scatter matrix which may render the solution less discriminative. *Second*, we further propose an incremental learning algorithm for sequential model update at new scene and/or dynamic deployments without model re-training from scratch. *Finally*, we investigate active sampling for more cost-effective re-id model update.

**Subspace Learning** The IRS is a discriminative subspace learning method, similar to distance metric learning (Yang and Jin 2006), Fisher discriminant analysis (FDA) (Fisher 1936; Fukunaga 2013), and cross-modal feature matching (Hardoon et al. 2007; Sharma et al. 2012; Kang et al. 2015). Representative metric learning re-id methods include PRDC (Zheng et al. 2013), KISSME (Koestinger et al. 2012), XQDA (Liao et al. 2015), MLAPG (Liao and Li 2015), LADF (Li et al. 2013), and so forth. PRDC maximises the likelihood of matched pairs with smaller distances than unmatched ones. KISSME measures the probability similarity of intra-class and inter-class feature differences under the Gaussian distribution assumption. sharing the spirit of Bayesian face model (Moghaddam et al. 2000). KISSME and Bayesian face are inefficient given high-dimensional features. XQDA overcomes this limitation by uniting dimension reduction and metric learning. MLAPG tackles the efficiency weakness in learning Mahalanobis function. While achieving significant performance gains, these methods focus *only* on one-time batch-wise model learning while ignore incremental learning capability. Our model is designed to fill this gap.

**Incremental Learning** Incremental learning (IL) concerns model training from data streams (Poggio and Cauwenberghs 2001). Often, IL requires extra immediate on-line model update for making the model ready to accept new data at any time. IL has been explored in many different vision tasks, e.g. image classification (Lin et al. 2011; Ristin et al. 2014). The closest works w.r.t. our model are three re-id methods (Liu et al. 2013; Wang et al. 2016c; Martinel et al. 2016).

Specifically, Liu et al. (2013) consider to optimise an error-prone post-rank search for refining quickly the ranking lists. this method is inherently restricted and unscalable due to the need for human feedback on all probe images independently. Wang et al. (2016c) solves this limitation by learning incrementally a unified generalisable re-id model from all available human feedback. Martinel et al. (2016) similarly consider incremental model update in deployment for maintaining re-id performance over time. Compared to these IL re-id methods, the IRS is uniquely characterised with more efficient optimisation (i.e. a closed-form solution) with the capability of low response latency. This is made possible by casting re-id model learning as a regression problem in the concept of well-design identity embedding space, in contrast to classification (Liu et al. 2013), verification (Martinel et al. 2016), or ranking (Prosser et al. 2010; Wang et al. 2016c) learning problem. Given that all these methods adopt their respective human verification designs and incremental learning strategies under distinct evaluation settings, it is impossible to conduct quantitative evaluation among them.

**Active Learning** Active learning (AL) is a strategy for reducing human labelling effort by selecting most informative samples for annotation (Settles 2012; Kang et al. 2004). Despite extensive AL studies on generic object classification (Osugi et al. 2005; Cebron and Berthold 2009; Hospedales et al. 2012; Ebert et al. 2012; Loy et al. 2012; Käding et al. 2015; Wang et al. 2016e), there exist little re-id attempts with only two works to our knowledge: active person identification (Das et al. 2015) and temporal re-id adaptation (Martinel et al. 2016).

Specifically, Das et al. (2015) learn a multi-class classifier on known identity classes for recognising training classes, therefore not a re-id model. Moreover, this model cannot support efficient incremental learning as Martinel et al. (2016) and IRS, due to expensive re-training from scratch and hence less suitable for AL with human in the loop. Martinel et al. (2016) explore also AL for incremental re-id model update. In comparison, our AL algorithm is more extensive and comprehensive (exploitation and exploration vs. exploitation alone) with better learning efficiency (no need for iterative optimisation and graph based data clustering). IRS is thus more suitable for human-in-the-loop driven incremental learning.

**Ridge Regression** Ridge regression (Hoerl and Kennard 1970; Hastie et al. 2005) is one of the most-studied learning algorithms. It has an efficient closed-form solution. with exiting optimised algorithms (Paige and Saunders 1982) readily applicable to large sized data. We ground the IRS re-id model on ridge regression for inheriting the learning efficiency and scalability advantages, Existing attempts for identity verification problems by class-label regression include (Liao et al. 2014; Sharma et al. 2012; Kang et al. 2015). Liao et al. (2014) adopted a linear regression based discriminant analysis method for re-id. Sharma et al. (2012) and Kang et al. (2015) proposed locality regularised class-label regression methods for recognition and retrieval.

Beyond these existing works, we systematically explore different label coding methods, non-linear regression kernelisation, model efficiency enhancement and labelling effort minimisation in an under-studied incremental re-id learning setting. Moreover, we bridge ridge regression and FDA (Fisher 1936; Fukunaga 2013) in feature embedding space design for more discriminatively encoding identity sensitive information. While the relationship between FDA and linear regression has been studied for binary-class (Duda et al. 2012) and multi-class (Hastie et al. 2005; Park and Park 2005) classification, this is the first study that formulates the two jointly in a single framework for person re-id.

**Data Scarcity** There are other generic approaches to solving the SSS challenge. Two common schemes are domain transfer (Layne et al. 2013; Ma et al. 2013; Peng et al. 2016; Geng et al. 2016; Li et al. 2017) and data augmentation (synthesis) (McLaughlin et al. 2015; Zheng et al. 2017). The former relies on auxiliary data (e.g. ImageNet or other re-id datasets) while the latter generates additional training data both for enriching the discriminative information accessible to model training. Conceptually, they are complementary to the proposed IRS with the focus on learning a more discriminative embedding space on the given training data from either scratch or pre-trained models. As shown in our evaluation, these approaches can be jointly deployed for further improving model generalisation (Table 9).

**Fig. 2:**
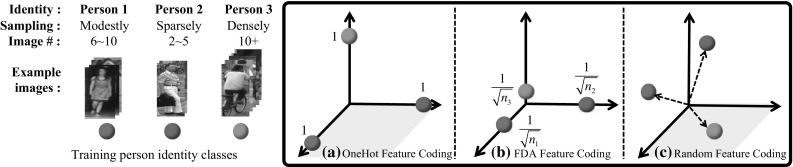
Illustration of feature embedding spaces obtained by three training class coding methods. Note, $n_i$ in (**b**) refers to the training image number of person *i* extracted from any cameras

## Identity Regression

### Problem Definition

We consider the image-based person re-identification problem (Gong et al. 2014). The key is to overcome the unconstrained person appearance variations caused by significant discrepancy in camera viewing condition and human pose (Fig. 1). To this end, we aim to formulate a feature embedding model for effectively and efficiently discovering identity discriminative information of cross-view person images.

Formally, we assume a labelled training dataset $\varvec{X} = [\varvec{x}_1,\ldots , \varvec{x}_i, \ldots , \varvec{x}_n] \in {\mathbb {R}}^{d\times n}$ where $\varvec{x}_i \in {\mathbb {R}}^{d \times 1}$ denotes the *d*-dimensional feature vector of image $\varvec{x}_i$, with the corresponding identity label vector $\varvec{l} = [l_1,\ldots , l_i,\ldots , l_n] \in {\mathbb {Z}}^{1 \times n}$, where $l_i \in \{1,\ldots ,c\}$ represents the identity label of image $\varvec{x}_i$ among a total of *c* identities. So, these *n* training images describe *c* different persons captured under multiple camera views. We omit the camera label here for brevity. The model learning objective is to obtain a discriminative feature embedding $\varvec{P} \in {\mathbb {R}}^{d\times m}$, i.e. in the embedding space, the distance between intra-person images is small whilst that of inter-person images is large regardless of their source camera views. In most existing works, the above criterion of compressing intra-person distributions and expanding inter-person distributions is encoded as classification/verification/ranking losses and then a feature embedding is learned by optimising the corresponding objective formulation. However, due to the SSS problem, the learned embedding space is often suboptimal and less discriminative. Also, there is often no clear interpretation on the learned embedding space.

Our method is significantly different: Prior to the model training, we first explicitly define an *ideal feature embedding space*, and then train a regression from the raw feature space to the defined embedding space. The learned regression function is our discriminative feature embedding. Specifically, we define a set of “*ideal*” target vectors in the embedding space, denoted by $\varvec{Y} = [\varvec{y}_1^\top ,\ldots ,\varvec{y}_n^\top ]^\top \in {\mathbb {R}}^{n \times m}$, and explicitly assign them to each of the training sample $\varvec{x}_i$, with $\varvec{y}_i \in {\mathbb {R}}^{1 \times m}$ referring to $\varvec{x}_i$’s target point in the feature embedding space, $i \in \{1,2,\ldots ,n\}$ and *m* referring to the feature embedding space dimension. In model training, we aim to obtain an optimal feature embedding $\varvec{P}$ that transforms the image feature $\varvec{x}$ into its mapping $\varvec{y}$ with labelled training data $\varvec{X}$. During model deployment, given a test probe image $\tilde{\varvec{x}}^p$ and a set of test gallery images $\{\tilde{\varvec{x}}_i^g\}$, we first transform them into the embedding space with the learned feature embedding $\varvec{P}$, denoted as $\tilde{\varvec{y}}^p$ and $\{\tilde{\varvec{y}}_i^g\}$ respectively. Then, we compute the pairwise matching distances between $\tilde{\varvec{y}}^p$ and $\{\tilde{\varvec{y}}_i^g\}$ by the Euclidean distance metric. Based on matching distances, we rank all gallery images in ascendant order. Ideally, the true match of the probe person is supposed to appear among top ranks.

### Identity Regression Space

To learn an optimal regression function as feature embedding, one key question in our framework is how to design the target “*ideal*” embedding space, in other words, how to set $\varvec{Y}$. We consider two principles in designing distribution patterns of training samples in the embedding space:

1. *Compactness* This principle concerns image samples belonging to the *same person class*. Even though each person’s intra-class distributions may be different in the raw feature space, we argue that in an optimal embedding space for re-id, the variance of all intra-class distributions should be suppressed. Specifically, for every training person, regardless of the corresponding sample size, all samples should be collapsed to a single point so that the embedding space becomes maximally discriminative with respect to person identity.
2. *Separateness* This principle concerns image samples belonging to the *different person classes*. Intuitively, the points of different person identities should be maximally separated in the embedding space. With a more intuitive geometry explanation, these points should be located on the vertices of a regular simplex with equal-length edges, so that the embedding space treats equally any training person with a well-separated symmetric structure.

Formally, we assign a unit-length vector on each dimension axis in the feature embedding space to every training person identity, i.e. we set $\varvec{y}_i = [y_{i,1},\ldots , y_{i,m}]$ for the *i*th training person (Fig. 2a) as:

1 $$\begin{aligned} {y}_{i,j} = {\left\{ \begin{array}{ll} 1, &{} \text{ if } \;\; l_i=j; \\ 0, &{} \text{ if } \;\; l_i\ne j. \end{array}\right. } \quad \text{ with } \quad j \in [1,2,\ldots ,m], \end{aligned}$$

where $l_i$ is the identity label of image $\varvec{x}_i$. We name this way of setting $\varvec{Y}$ as *OneHot Feature Coding*. The embedding space defined by Eq. (1) has a few interesting properties:

1. Each dimension in the embedding space corresponds to one specific training person’s identity.
2. Training persons are evenly distributed in the embedding space and the distances between any two training persons are identical.
3. Geometrically, the points of all training person identities together form a standard simplex.

Because each dimension of this embedding space can be now interpreted by one specific training identity, we call such an embedding space an *identity regression space*. Having the identity regression space defined by Eq. (1), we propose to exploit the multivariate ridge regression algorithm (Hoerl and Kennard 1970; Zhang et al. 2010).

**Fig. 3:**
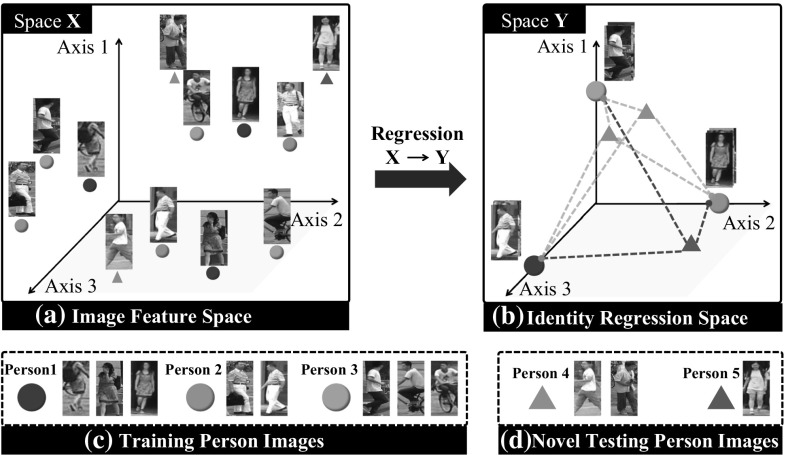
Illustration of our Identity Regression Space (IRS) person re-id model. During model training, by regression we learn an identity discriminative feature embedding from (**a**) the image feature space to (**b**) the proposed identity regression space defined by (**c**) all training person classes (indicated by circles). During deployment, we can exploit the learned feature embedding to re-identify **d** novel testing person identities (indicated by triangles) in IRS

In particular, by treating $\varvec{Y}$ as the regression output and $\varvec{P}$ as the to-be-learned parameter, we search for a discriminative projection by minimising the mean squared error as:

2 $$\begin{aligned} \varvec{P}^* = \arg \min _{\varvec{P}} \; \frac{1}{2}\Vert \varvec{X}^\top \varvec{P} - \varvec{Y}\Vert _F^2\,+\,\lambda \Vert \varvec{P}\Vert _F^2, \end{aligned}$$

where $\Vert \cdot \Vert _F$ is the Frobenius norm, $\lambda$ controls the regularisation strength. Critically, this formulation has an efficient closed-form solution:

3 $$\begin{aligned} \varvec{P}^* = \big (\varvec{X}\varvec{X}^\top \,+\,\lambda \varvec{I}\big )^\dagger \varvec{X}\varvec{Y}, \end{aligned}$$

where $(\cdot )^\dagger$ denotes the Moore–Penrose inverse, and $\varvec{I}$ the identity matrix. Since our model learning is by regression towards a training identity space, we call this method the “identity regression space” (IRS) model (Fig. 3). The IRS re-id feature learning requirement leads naturally to exploiting the ridge regression method for learning the mapping between image features and this semantic identity space. The novelty of this approach is not in Eq. (2) itself, but the IRS learning concept in the re-id context. Note that, we do not select deep models (Xiao et al. 2016) in our IRS implementation due to their intrinsic weakness for model incremental learning. Nevertheless, in our experiments we also evaluated IRS with a deep learning model (Sect. 5.1, IV and V). Technically, OneHot based IRS *feature coding* and *embedding* differs fundamentally from deep learning classification models due to two modelling differences: (1) Whilst the latter adopts one-hot *class label vectors*, the underlying optimised deep features (e.g. the feature layer outputs) are not of one-hot style, i.e. not an IRS embedding. (2) A single softmax prediction may correspond to multiple different logit (i.e. feature) inputs. Specifically, even if two logit inputs are different, as long as the corresponding element is *relatively* larger than others, both their softmax outputs will be close to the same one-hot vector. In other words, for deep classification models the underlying feature representations of each class are not unique. Therefore, deep classification model are trained under a weaker learning constraint than the IRS whose feature embedding is trained strictly with only one ground-truth feature vector per class. The regression algorithm selection is independent of the generic IRS concept.

#### Remark

Unlike Fisher discriminant analysis (Fisher 1936), the proposed IRS has no need for the intra-class and between-class scatter matrices. This renders our model more suitable for addressing the small sample size (SSS) problem since the intra-class scatter matrix of sparse training data will become singular, which results in computational difficulty (Fukunaga 2013). To solve this SSS problem, one straightforward approach is performing dimensionality reduction (e.g. principal component analysis) before model learning (Pedagadi et al. 2013). This however may cause the loss of discriminative power. An alternative method is directly rectifying the intra-class scatter by adding a non-singular regularisation matrix (Mika et al. 1999; Xiong et al. 2014; Liao et al. 2015). Nonetheless, both approaches as above suffer from the degenerate eigenvalue problem (i.e. several eigenvectors share the same eigenvalue), which makes the solution sub-optimal with degraded discrimination (Zheng et al. 2005). As a more principled solution, the null Foley–Sammon transform (NFST) modifies the Fisher discriminative criterion—Finding null projecting directions on which the intra-class distance is zero whilst the between-class distance is positive—so that more discriminant projections corresponding to the infinitely large Fisher criterion can be obtained (Chen et al. 2000; Guo et al. 2006). The NFST has also been recently employed to solve the SSS problem in re-id (Zhang et al. 2016a). While reaching the largest Fisher objective score via exploiting the null space of intra-class scatter matrix by NFST, the between-class scatter is not maximised and therefore still an incomplete Fisher discriminative analysis. It is easy to see that the proposed IRS model shares the spirit of NFST in terms of projecting same-class images into a single point in order to achieve the extreme class *compactness* and most discriminative feature embedding. However, unlike the NFST’s positive between-class scatter constraint—a weaker optimisation constraint likely resulting in lower discriminative power, the model proposed here optimises instead the between-class *separateness* by enforcing the orthogonality between any two different person classes in the target feature space to maximise the class discrimination and separation in a stronger manner. In terms of model optimisation, we resort to the more efficient ridge regression paradigm rather than the Fisher criterion. Overall, we consider that our IRS conceptually extends the NFST by inheriting its local compact classes merit whilst addressing its global class distribution modelling weakness in a more efficient optimisation framework. In our evaluations, we compare our IRS model with the NFST and show the advantages from this new formulation in terms of both model efficiency and discriminative power.

**Alternative Feature Coding** Apart from the OneHot feature coding (Eq. 1), other designs of the embedding space can also be readily incorporated into our IRS model. We consider two alternative feature coding methods. The first approach respects the Fisher discriminant analysis (FDA) (Fisher 1936; Fukunaga 2013) criterion, named *FDA Feature Coding*, which is adopted in the preliminary version of this work (Wang et al. 2016b). Formally, the FDA criterion can be encoded into our IRS model by setting target identity regression space as (Fig. 2b):

4 $$\begin{aligned} {y}_{ij} = {\left\{ \begin{array}{ll} \frac{1}{\sqrt{n_i}}, &{} \text{ if } \;\; l_i=j; \\ 0, &{} \text{ if } \;\; l_i\ne j. \end{array}\right. } \quad \text{ with } \quad j \in [1,2,\ldots ,m]. \end{aligned}$$

where $n_i$ and $l_i$ refers to the total image number and identity label of training person *i*. A detailed derivation is provided in “Appendix”. As opposite to Eq. (1) which treats each person identity equally (e.g. assigning them with unit-length vectors in the embedding space), this FDA coding scheme assigns variable-length vectors with the length determined by $n_i$. As shown in (Fig. 2b), with the FDA criterion, the resulting training identity simplex in the embedding space is no longer regular. This may bring benefits for typical classification problems by making size-sensitive use of available training data for modelling individual classes as well as possible, but not necessarily for re-id. Particularly, modelling training classes in such a biased way may instead hurt the overall performance since the re-id model is differently required to generalise the knowledge from training person classes to previously unseen testing ones other than within the training ones as in conventional classification.

**Fig. 4:**
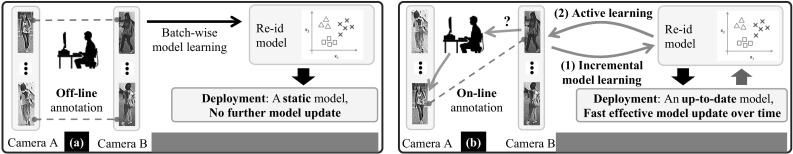
Illustration of different person re-id model learning settings. **a** Batch-wise person re-id model learning: a re-id model is first learned on an exhaustively labelled training set, and then fixed for deployment without model update. **b** Incremental person re-id model learning: Training samples are collected sequentially on-the-fly with either random or active unlabelled data selection, and the re-id model keeps up-to-date by efficient incremental learning from the newly labelled data over time

The second alternative is *Random Feature Coding*. That is, we allocate for each training identity a *m*-dimensional random vector with every element following a uniform distribution over the range of [0, 1] (Fig. 2c). Random coding has shown encouraging effect in shape retrieval (Zhu et al. 2016) and face recognition (Zhang et al. 2013). In this way, individual dimensions are no longer identity-specific and training identity regression space are shared largely irregularly. We will evaluate the effectiveness of these three feature coding methods in Sect. 5.1.

### Kernelisation

Given complex variations in viewing condition across cameras, the optimal subspace may not be obtainable by linear projections. Therefore, we further kernelise the IRS model (Eq. 3) by projecting the data from the original visual feature space into a reproducing kernel Hilbert space ${\mathcal {H}}$ with an implicit feature mapping function $\phi (\cdot )$. The inner-product of two data points in ${\mathcal {H}}$ can be computed by a kernel function: $h_\text {k}(\varvec{x}_i, \varvec{x}_j) = \left\langle \phi (\varvec{x}_i), \phi (\varvec{x}_j) \right\rangle$. By $h_\text {k}$ (we utilised the typical RBF or Gaussian kernel in our implementation), we obtain a kernel representation $\varvec{K}\in {\mathbb {R}}^{n\times n}$, based on which a corresponding non-linear projection solution can be induced as:

5 $$\begin{aligned} \varvec{Q}^* = \big ( \varvec{K}\varvec{K}^\top \,+\,\lambda \varvec{K} \big )^\dagger \varvec{K}\varvec{Y}. \end{aligned}$$

Once test samples are transformed into the kernel space with $h_k$, we can similarly apply the learned projection $\varvec{Q}^*$ as the linear case. We use the kernel version throughout all experiments due to its capability of modelling the non-linearity which is critical for open space re-id in images with complex person appearance variations across camera views.

## Incremental Identity Regression

In Sect. 3, we presented the proposed IRS person re-id model. Similar to the majority of conventional re-id methods, we assume a batch-wise model learning setting: First collecting all labelled training data and then learning the feature embedding model (Fig. 4a). In real-world scenario, however, data annotation is likely to arrive in sequence rather than at one time particularly when deployed to new arbitrary scenes. In such case, a practical system requires the incremental learning capability for cumulatively learning and updating the re-id model over deployment process (Fig. 4b-(1)). On the other hand, incremental learning is essential for temporal model adaptation, e.g. handling the dynamics in the deployment context (Martinel et al. 2016). A simple and straightforward scheme is to re-train the model from scratch using the entire training dataset whenever any newly labelled samples become available. Obviously, this is neither computational friendly nor scalable particularly for resource/budget restricted deployment.

To overcome this limitation, we introduce an incremental learning algorithm, named IRS$^\text {inc}$, for enabling fast model update without the need for re-training from scratch. Suppose at time *t*, we have the feature matrix $\varvec{X}_t \in {\mathbb {R}}^{d\times n_t}$ of $n_t$ previously labelled images of $c_t$ person identities, along with $\varvec{Y}_t \in {\mathbb {R}}^{n_t \times m}$ their indicator matrix defined by Eq. (1). We also have the feature matrix $\varvec{X}' \in {\mathbb {R}}^{d\times n'}$ of $n'$ newly labelled images of $c'$ new person classes, with $\varvec{Y}' \in {\mathbb {R}}^{n' \times (c_t\,+\,c')}$ the corresponding indicator matrix similarly defined by Eq. (1). After merging the new data, the updated feature and identity embedding matrix can be represented as:

6 $$\begin{aligned} \varvec{X}_{t\,+\,1} = [\varvec{X}_t, \; \varvec{X}'], \quad \varvec{Y}_{t\,+\,1} = \Big [ \begin{array}{c} \varvec{Y}_t\oplus \varvec{0}\\ \varvec{Y}' \end{array} \Big ], \end{aligned}$$

where $(\cdot )\oplus \varvec{0}$ denotes the matrix augmentation operation, i.e. padding an appropriate number of zero columns on the right. By defining

7 $$\begin{aligned} \varvec{T}_t = \varvec{X}_t\varvec{X}_t^\top , \end{aligned}$$

and applying Eq. (6), we have

8 $$\begin{aligned} \varvec{T}_{t\,+\,1} = \varvec{T}_t\,+\,\varvec{X}'\varvec{X}'^{\top }. \end{aligned}$$

For initialisation, i.e. when $t=0$, we set $\varvec{T}_0 = \varvec{X}_0\varvec{X}_0^\top \,+\,\lambda \varvec{I}$. Also, we can express the projection $\varvec{P}_t \in {\mathbb {R}}^{d \times m}$ (Eq. 3) of our IRS model at time *t* as

9 $$\begin{aligned} \varvec{P}_{t} = \varvec{T}_{t}^\dagger \varvec{X}_{t} \varvec{Y}_{t}. \end{aligned}$$

Our aim is to obtain the feature embedding $\varvec{P}_{t\,+\,1}$, which requires to compute $\varvec{T}_{t\,+\,1}^{\dagger }$. This can be achieved by applying the Sherman–Morrison–Woodbury formula (Woodbury 1950) to Eq. (8) as:

10 $$\begin{aligned} \varvec{T}_{t\,+\,1}^{\dagger } = \varvec{T}_t^\dagger - \varvec{T}_t^\dagger \varvec{X}'\big (\varvec{I}\,+\,\varvec{X}'^{\top }\varvec{T}_t^\dagger \varvec{X}'\big )^\dagger \varvec{X}'^{\top }\varvec{T}_t^\dagger . \end{aligned}$$

Equations (3) and (6) together give us:

11 $$\begin{aligned} \varvec{P}_{t\,+\,1}&= \varvec{T}_{t\,+\,1}^\dagger \varvec{X}_{t\,+\,1} \varvec{Y}_{t\,+\,1} \nonumber \\&= (\varvec{T}_{t\,+\,1}^\dagger \varvec{X}_t \varvec{Y}_t)\oplus \varvec{0} \,+\,\varvec{T}_{t\,+\,1}^\dagger \varvec{X}'\varvec{Y}'. \end{aligned}$$

Further with Eqs. (10) and (9), we can update $\varvec{P}$ as:

12 $$\begin{aligned} \varvec{P}_{t\,+\,1}&= \Big (\varvec{P}_t - \varvec{T}_t^\dagger \varvec{X}'\big (\varvec{I}\,+\,\varvec{X}'^{\top }\varvec{T}_t^\dagger \varvec{X}'\big )^\dagger \varvec{X}'^{\top }\varvec{P}_t\Big ) \oplus \varvec{0}\nonumber \\&\quad \,+\,\varvec{T}_{t\,+\,1}^\dagger \varvec{X}'\varvec{Y}'. \end{aligned}$$

Note, the model update (Eqs. 10, 12) only involves newly coming data samples. Hence, our method does not require to store the training data once used for model update. As only cheap computational cost is involved in such linear operations, the proposed algorithm well suits for on-line responsive re-id model learning and updating in deployment at large scales in reality.

**Implementation Consideration** The IRS$^\text {inc}$ model supports incremental learning given either a single new sample ($n' = 1$) or a small chunk of new samples ($n' \geqslant 2$). If the data chunk size $n'\ll d$ (where *d* is the feature dimension), it is faster to perform $n'$ separate updates on each new sample instead of by a whole chunk. The reason is that, in such a way the Moore–Penrose matrix inverse in Eqs. (10) and (12) can be reduced to $n'$ separate scaler inverse operations, which is much cheaper in numerical computation.

### Active Learning for Cost-Effective Incremental Update

The incremental learning process described above is *passive*, i.e. a human annotator is supposed to label randomly chosen data without considering the potential value of each selected sample in improving the re-id model. Therefore, data annotation by this random way is likely to contain redundant information with partial labelling effort wasted. To resolve this problem, we explore the active learning idea (Settles 2012) for obtaining more cost-effective incremental re-id model update (Fig. 4b-(2)).

**Active IRS**$^\text {inc}$ **Overview.** In practice, we often have access to a large number of *unlabelled* images $\widetilde{\mathcal {P}}$ and $\widetilde{\mathcal {G}}$ captured by disjoint cameras. Assume at time step $t \in \{1,\ldots ,\tau \}$ with $\tau$ defining the pre-determined human labelling budget, we have the up-to-date IRS$^\text {inc}$ model $m_t$ (corresponding to the feature embedding $\varvec{P}_t$), along with $\widetilde{\mathcal {P}_t}$ and $\widetilde{\mathcal {G}_t}$ denoting the remaining unlabelled data. To maximise labelling profit, we propose an *active labelling* algorithm for IRS$^\text {inc}$ with the main steps as follows:

1. An image $\varvec{x}_t^p \in \widetilde{\mathcal {P}_t}$ of a new training identity $l_t$ is *actively* selected by model $m_t$, according to its potential usefulness and importance measured by certain active sampling criteria (see details below).
2. A ranking list of unlabelled images $\widetilde{\mathcal {G}}_t$ against the selected $\varvec{x}_t^p$ is then generated by $m_t$ based matching distances.
3. For the selected $\varvec{x}_t^p$, a human annotator is then asked to manually identify the cross-view true matching image $\varvec{x}_t^g \in \widetilde{\mathcal {G}}_t$ in the ranking list, and then generate a new annotation ($\varvec{x}_t^p, \varvec{x}_t^g$).
4. The IRS$^\text {inc}$ re-id model is updated to $m_{t\,+\,1}$ (i.e. $\varvec{P}_{t\,+\,1}$) from the new data annotation $(\varvec{x}_t^p, \varvec{x}_t^g)$ by our incremental learning algorithm (Eqs. 10, 12).

**Fig. 5:**
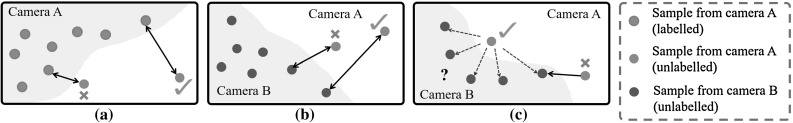
Illustration of the proposed active exploration and exploitation selection criteria for more cost-effective incremental re-id model learning. **a** Appearance diversity. **b** Matching discrepancy. **c** Ranking uncertainty

Among these steps above, the key lies in how to select a good image $\varvec{x}_t^p$. To this end, we derive a “Joint Exploration–Exploitation” (**JointE**$^2$) active sampling algorithm composed of three criteria as follows (Fig. 5).

**(I) Appearance Diversity Exploration** Intuitively, the appearance diversity of training people is a critical factor for the generalisation capability of a re-id model. Thus, the preferred next image to annotate should lie in the most unexplored region of the population $\widetilde{\mathcal {P}_t}$. Specifically, at time *t*, the distance between any two samples $(\varvec{x}_1,\varvec{x}_2)$ by the current re-id model is computed as:

13 $$\begin{aligned} d(\varvec{x}_1,\varvec{x}_2|m_t) = (\varvec{x}_1 - \varvec{x}_2)^\top \varvec{P}_t\varvec{P}^\top _t(\varvec{x}_1 - \varvec{x}_2). \end{aligned}$$

Given the unlabelled $\widetilde{\mathcal {P}}_t$ and labelled $\mathcal {P}_t$ part of the set $\widetilde{\mathcal {P}}$ ($\widetilde{\mathcal {P}}_t \bigcup \mathcal {P}_t = \widetilde{\mathcal {P}}$), we can measure the diversity degree of an unlabelled sample ${\varvec{x}}_i^p \in \widetilde{\mathcal {P}}_t$ by its distance against the *within-view nearest neighbour* in $\mathcal {P}_t$ (Fig. 5a):

14 $$\begin{aligned} \begin{aligned} \varepsilon _1({\varvec{x}}_i^p) = \min \; d({\varvec{x}}_i^p, \varvec{x}_j^p|m_t), \\ \text{ s.t. } \;\; {\varvec{x}}_i^p \in \widetilde{\mathcal {P}}_t, \;\; \varvec{x}_j^p \in \mathcal {P}_t. \end{aligned} \end{aligned}$$

Equation (14) defines the distance of an *unlabelled* sample $\varvec{x}_i^p$ from the labelled set, i.e. the distance between $\varvec{x}_i^p$ and its nearest labelled sample. This is not an optimisation operation. It is a nearest sample search by “min” operation. By maximising the nearest distances, more diverse person appearance can be covered and learned for more rapidly increasing the knowledge of the IRS$^\text {inc}$ model, avoiding repeatedly learning visually similar training samples.

**(II) Matching Discrepancy Exploration** A well learned re-id model is supposed to find the true match of a given image with a small cross-view matching distance. In this perspective, our second criterion particularly prefers the samples with large matching distances in the embedding space, i.e. the re-id model $m_t$ remains largely unclear on what are the likely corresponding cross-view appearances of these “unfamiliar” people. Numerically, we compute the matching distance between an unlabelled sample ${\varvec{x}}_i^p \in \widetilde{\mathcal {P}}_t$ and the cross-view true match (assumed as *cross-view nearest neighbour*) in $\widetilde{\mathcal {G}}$ (Fig. 5b):

15 $$\begin{aligned} \varepsilon _2({\varvec{x}}_i^p)&= \min \; d({\varvec{x}}_i^p, \varvec{x}_j^g|m_t), \nonumber \\&\text{ s.t. } \;\; {\varvec{x}}_i^p \in \widetilde{\mathcal {P}}_t, \;\; \varvec{x}_j^g \in \widetilde{\mathcal {G}}. \end{aligned}$$

That is, the unlabelled images with greater $\varepsilon _2({\varvec{x}}_i^p)$ are preferred to be selected.

**(III) Ranking Uncertainty Exploitation** Uncertainty-based exploitative sampling schemes have been widely investigated for classification problems (Joshi et al. 2009; Settles and Craven 2008; Ebert et al. 2012). The essential idea is to query the least certain sample for human to annotate. Tailored for re-id tasks with this idea, given the similar appearance among different identities, a weak re-id model may probably generate similar ranking scores for those visually ambiguous gallery identities with respect to a given probe. Naturally, it should be useful and informative to manually label such “challenging” samples for enhancing a person re-id model’s discrimination power particularly with regard to such person appearance (Fig. 5c). To obtain such person images, we define a matching distance based probability distribution over all samples $\varvec{x}^g_j \in \widetilde{\mathcal {G}}$ for a given cross-view image $\varvec{x}^p_i \in \widetilde{\mathcal {P}}$:

16 $$\begin{aligned} p_{m_t}(\varvec{x}^g_j|\varvec{x}^p_i) = \frac{1}{Z_{i}^t}{e^{-d({\varvec{x}}_i^p, \varvec{x}_j^g|m_t)}}, \end{aligned}$$

where

$$\begin{aligned} Z_{i}^t = {\sum _k e^{-d({\varvec{x}}_i^p, \varvec{x}_k^g|m_t)}}, \;\; \varvec{x}_k^g \in \widetilde{\mathcal {G}}. \end{aligned}$$

The quantity $p_{m_t}(\varvec{x}^g_j|\varvec{x}^p_i)$ gives a high entropy when most ranking scores are adjacent to each other, indicating great information to mine from the perspective of information theory (Akaike 1998). In other words, the model has only a low confidence on its generated ranking list considering that only a very few number of cross-camera samples are likely to be true matches rather than many of them. Consequently, our third criterion is designed as:

17 $$\begin{aligned} \varepsilon _3({\varvec{x}}_i^p)&= - \sum _j p_{m_t}\left( \varvec{x}^g_j|\varvec{x}^p_i\right) \log p_{m_t}\left( \varvec{x}^g_j|\varvec{x}^p_i\right) ,\nonumber \\&\quad \text{ s.t. } \;\; {\varvec{x}}_i^p \in \widetilde{\mathcal {P}}_t, \;\; \varvec{x}_j^g \in \widetilde{\mathcal {G}}. \end{aligned}$$

which aims to select out those associated with high model ranking ambiguity.
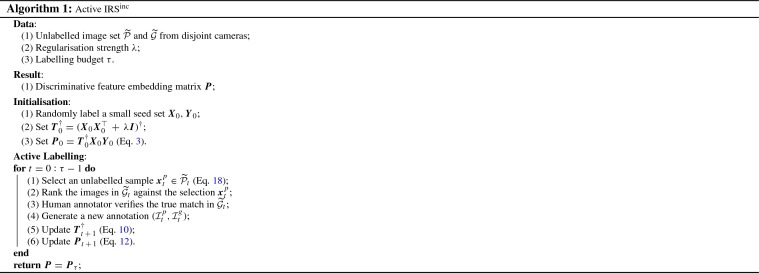

**Table 1:** Statistics of person re-id datasets

Dataset	Cameras	Persons	Labelled BBox	Detected BBox
VIPeR	2	632	1264	0
CUHK01	2	971	1942	0
CUHK03	6	1467	14,097	14,097
Market-1501	6	1501	0	32,668

*BBox* bounding box

**Fig. 6:**
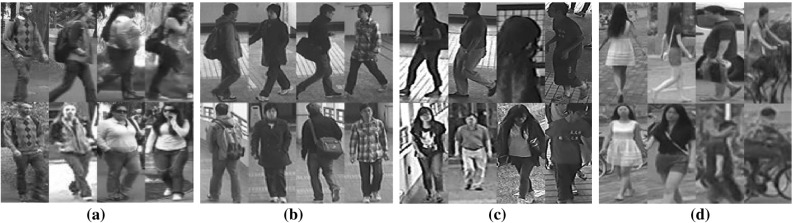
Example person images from four person re-id datasets. Two images of each individual columns present the same person. **a** VIPeR(Gray et al. 2007). **b** CUHK01 (Li et al. 2012). **c** CUHK03 (Li et al. 2014) and **d** Market-1501 (Zheng et al. 2015)

**Joint Exploration–Exploitation** Similar to the model in (Cebron and Berthold 2009; Ebert et al. 2012), we combine both exploitation and exploration based criteria into our final active selection standard, formally as:

18 $$\begin{aligned} \varepsilon \left( \varvec{x}^p_i\right) = \varepsilon _1\left( \varvec{x}^p_i\right) \,+\,\varepsilon _2\left( \varvec{x}^p_i\right) \,+\,\varepsilon _3\left( \varvec{x}^p_i\right) . \end{aligned}$$

To eliminate scale discrepancy, we normalise $\varepsilon _1, \varepsilon _2, \varepsilon _3$ to the unit range [0, 1] respectively before fusing them. Specifically, given $\varepsilon _1$ scores of all unlabelled samples, we normalise them by dividing the maximal value so that the highest $\varepsilon _1$ is 1. The same operation is performed on $\varepsilon _2$ and $\varepsilon _3$.

In summary, with Eq. (18), all the unlabelled samples in $\widetilde{\mathcal {P}}$ can be sorted accordingly, and the one with highest $\varepsilon (\varvec{x}^p_i)$ is then selected for human annotation. An overview of our proposed active learning based incremental model learning and updating is presented in Algorithm 1. We will show the effect of our proposed active labelling method in our evaluations (Sect. 5.2).

### Kernelisation

We kernelise similarly the incremental IRS algorithm as in Sect. 3.3. Specifically, we first obtain the kernel representation of new training data and then conduct model incremental learning in the Hilbert space. We utilise the kernelised model with its non-linear modelling power in all incremental re-id model learning experiments including active sampling with human-in-the-loop.

## Experiments

**Datasets** For model evaluation, four person re-id benchmarks were used: VIPeR (Gray et al. 2007), CUHK01 (Li et al. 2012), CUHK03 (Li et al. 2014), and Market-1501 (Zheng et al. 2015), as summarised in Table 1. We show in Fig. 6 some examples of person images from these datasets. Note that the datasets were collected with different data sampling protocols: (a) VIPeR has one image per person per view; (b) CUHK01 contains two images per person per view; (c) CUHK03 consists of a maximum of five images per person per view, and also provides both manually labelled and auto-detected image bounding boxes with the latter posing more challenging re-id test due to unknown misalignment of the detected bounding boxes; (d) Market-1501 has variable numbers of images per person per view. These four datasets present a good selection of re-id test scenarios with different population sizes under realistic viewing conditions exposed to large variations in human pose and strong similarities among different people.

**Features** To capture the detailed information of person appearance, we adopted three state-of-the-art feature representations with variable dimensionalities from 10$^4$ to 10$^2$: (**1**) *Local Maximal Occurrence* (LOMO) feature (Liao et al. 2015): The LOMO feature is based on a HSV colour histogram and Scale Invariant Local Ternary Pattern (Liao et al. 2010). For alleviating the negative effects caused by camera view discrepancy, the Retinex algorithm (Land and McCann 1971) is applied to pre-process person images. The feature dimension of LOMO is rather high at 26,960, therefore expensive to compute.

(**2**) *Weighted Histograms of Overlapping Stripes* (WHOS) feature (Lisanti et al. 2014, 2015): The WHOS feature contains HS/RGB histograms and HOG (Wang et al. 2009) of image grids, with a centre support kernel as weighting to approximately segmented person foreground from background clutters. We implemented this feature model as described by Lisanti et al. (2014). The feature dimension of WHOS is moderate at 5138.

(**3**) *Convolutional Neural Network* (CNN) feature (Xiao et al. 2016): Unlike hand-crafted LOMO and WHOS features, deep CNN person features are learned from image data. Specifically, we adopted the DGD CNN (Xiao et al. 2016) and used the FC$_7$ output as re-id features. The DGD feature has a rather low dimension of 256, thus efficient to extract. Following Xiao et al. (2016), we trained the DGD by combining labelled and detected person bounding box images (a total 26,246 images) with the original authors released codes. We then deployed the trained DGD to extract deep features of the test image data for CUHK03 (the same domain). On Market-1501, the CUHK03 trained DGD was further fine-tuned on the 12,936 Market-1501 training images for domain adaptation. On VIPeR and CUHK01, the CUHK03 trained DGD was *directly* deployed *without* any fine-tuning as there are insufficient training images to make effective model adaptation, with only 632 and 1940 training images for VIPeR and CUHK01 respectively.

**Model Training Settings** In evaluations, we considered extensively comparative experiments under two person re-id model training settings: **(I)** *Batch-wise model training*: In this setting, we followed the conventional supervised re-id scheme commonly utilised in most existing methods, that is, first collecting all training data and then learning a re-id model *before* deployment. **(II)** *Incremental model training*: In contrast to the batch-wise learning, we further evaluated a more realistic data labelling scenario where more training labels are further collected over time *after* model deployment. The proposed IRS$^{\text {inc}}$ model was deployed for this incremental learning setting.

### Batch-Wise Person Re-id Evaluation

**Batch-Wise Re-id Evaluation Protocol** To facilitate quantitative comparisons with existing re-id methods, we adopted the standard supervised re-id setting to evaluate the proposed IRS model. Specifically, on *VIPeR*, we split randomly the whole population of the dataset (632 people) into two halves: One for training (316) and another for testing (316). We repeated 10 trials of random people splits and utilised the averaged results. On *CUHK01*, we considered two benchmarking training/test people split settings: (1) 485/486 split: randomly selecting 485 identities for training and the other 486 for testing (Liao et al. 2015; Zhang et al. 2016a; 2) 871/100 split: randomly selecting 871 identities for training and the other 100 for testing (Ahmed et al. 2015; Shi et al. 2016). As CUHK01 is a multi-shot (e.g. multiple images per person per camera view) dataset, we computed the final matching distance between two people by averaging corresponding cross-view image pairs. Again, we reported the results averaged over 10 random trials for either people split. On *CUHK03*, following Li et al. (2014) we repeated 20 times of random 1260/100 people splits for model training/test and reported the averaged accuracies under the single-shot evaluation setting (Zhang et al. 2016a). On *Market-1501*, we used the standard training/test (750/751) people split provided by Zheng et al. (2015). On all datasets, we exploited the cumulative matching characteristic (CMC) to measure the re-id accuracy performance. On Market-1501, we also considered the recall measure of multiple truth matches by mean average precision (mAP), i.e. first computing the area under the precision-recall curve for each probe, then calculating the mean of average precision over all probes (Zheng et al. 2015).

In the followings, we evaluated: (i) Comparisons to state-of-the-arts, (ii) Effects of embedding space design, (iii) Effects of features, (iv) Deep learning regression, (v) Complementary of transfer learning and IRS, (vi) Comparisons to subspace/metric learning models, (vii) Regularisation sensitivity, and (viii) Model complexity.

**(I) Comparisons to the State-of-The-Arts** We first evaluated the proposed IRS model by extensive comparisons to the existing state-of-the-art re-id models under the standard supervised person re-id setting. We considered a wide range of existing re-id methods, including both hand-crafted and deep learning models. In the following experiments, we deployed the *OneHot Feature Coding* (Eq. 1 in Sect. 3.2) for the identity regression space embedding of our IRS model unless stated otherwise. We considered both single- and multi-feature based person re-id performance, and also compared re-id performances of different models on auto-detected person boxes when available in CUHK03 and Market-1501.

***Evaluation on VIPeR*** Table 2 shows a comprehensive comparison on re-id performance between our IRS model (and its variations) and existing models using the VIPeR benchmark (Gray et al. 2007). It is evident that our IRS model with a non-deep feature LOMO, IRS (LOMO), is better than all existing methods^1^ except the deep model MCP (Cheng et al. 2016), with Rank-1 45.1 versus 47.8% respectively. Interestingly, using our CUHK03 trained CNN deep feature *without* fine-tuning on VIPeR, i.e. IRS (CNN), does not offer extra advantage (Rank-1 33.1%), due to the significant domain drift between VIPeR and CUHK03. This becomes more clear when compared with the CUHK01 tests below. Moreover, given a score-level fusion on the matching of three different features, IRS (WHOS + LOMO + CNN), the IRS can benefit from further boosting on its re-id performance, obtaining the best Rank-1 rate at 54.6%. These results demonstrate the effectiveness of the proposed IRS model in learning identity discriminative feature embedding because of our *unique* approach on identity regression to learning a re-id feature embedding space, in contrast to existing established ideas on classification, verification or ranking based supervised learning of a re-id model.

**Table 2:** Re-id performance comparison on the VIPeR benchmark

Dataset	VIPeR			
Rank (%)	R1	R5	R10	R20
LADF (Li et al. 2013)	29.3	61.0	76.0	88.1
MFA (Yan et al. 2007)	32.2	66.0	79.7	90.6
kLFDA (Xiong et al. 2014)	38.6	69.2	80.4	89.2
XQDA (Liao et al. 2015)	40.0	68.1	80.5	91.1
MLAPG (Liao and Li 2015)	40.7	69.9	82.3	92.4
NFST (Zhang et al. 2016a)	42.3	71.5	82.9	92.1
LSSCDL (Zhang et al. 2016b)	42.7	–	84.3	91.9
TMA (Martinel et al. 2016)	43.8	–	83.8	91.5
HER (Wang et al. 2016b)	45.1	74.6	85.1	93.3
DML (Yi et al. 2014)	28.2	59.3	73.5	86.4
DCNN$+$ (Ahmed et al. 2015)	34.8	63.6	75.6	84.5
SICI (Wang et al. 2016a)	35.8	–	–	–
DGD (Xiao et al. 2016)	38.6	–	–	
Gated S-CNN (Varior et al. 2016a)	37.8	66.9	77.4	–
MCP (Cheng et al. 2016)	**47**.**8**	74.7	84.8	91.1
**IRS (WHOS)**	44.5	**75**.**0**	**86.3**	**93.6**
**IRS (LOMO)**	45.1	74.6	85.1	93.3
**IRS (CNN)**	33.1	59.9	71.5	82.2
MLF$^{\mathrm{a}}$ (Zhao et al. 2014)	43.4	73.0	84.9	93.7
ME$^{\mathrm{a}}$ (Paisitkriangkrai et al. 2015)	45.9	77.5	88.9	95.8
CVDCA$^{\mathrm{a}}$ (Chen et al. 2016c)	47.8	76.3	86.3	94.0
FFN-Net$^{\mathrm{a}}$ (Wu et al. 2016)	51.1	81.0	91.4	**96.9**
NFST$^{\mathrm{a}}$ (Zhang et al. 2016a)	51.2	82.1	90.5	95.9
HER$^{\mathrm{a}}$ (Wang et al. 2016b)	53.0	79.8	89.6	95.5
GOG$^{\mathrm{a}}$ (Matsukawa et al. 2016)	49.7	–	88.7	94.5
SCSP$^{\mathrm{a}}$ (Chen et al. 2016a)	53.5	**82.6**	**91.5**	96.7
**IRS (WHOS + LOMO + CNN)** $^{\mathrm{a}}$	**54**.**6**	81.5	90.3	95.7

Best results for single-feature and multi-feature are given in bold

$^{\mathrm{a}}$Multiple features fusion

***Evaluation on CUHK01*** Table 3 shows a comprehensive comparison of the IRS model with existing competitive re-id models on the CUHK01 benchmark (Li et al. 2012). It is clear that the proposed IRS model achieves the best re-id accuracy under both training/test split protocols. Note that, HER (Wang et al. 2016b) is IRS-FDA (LOMO). Specifically, for the 486/485 split, our IRS (CNN) method surpassed the deep learning DGD model (Xiao et al. 2016), the second best in this comparison, by Rank-1 $2.0\%\,(68.6{-}66.6)$. For the 871/100 split, IRS (CNN) yields a greater performance boost over DGD with improvement on Rank-1 at $12.6\%\,(84.4{-}71.8)$. It is also worth pointing out that the DGD model was trained using data from other 6 more datasets and further carefully fine-tuned on CUHK01. In contrast, our IRS (CNN) model was only trained on CUHK03 without fine-tuning on CUHK01, and the CNN architecture we adopted closely resembles to that of DGD. By fusing multiple features, the performance margin of IRS (WHOS + LOMO + CNN) over the existing models is further enlarged under both splits, achieving Rank-1 $11.7\%\,(80.8{-}69.1)$ boost over NFST (Zhang et al. 2016a) and Rank-1 $16.6\%\,(88.4{-}71.8)$ boost over SICI (Wang et al. 2016a), respectively. Compared to VIPeR, the overall re-id performance advantage of the IRS model on CUHK01 is greater over existing models. This is due to not only identity prototype regression based feature embedding, but also less domain drift from CUHK03 to CUHK01, given that the CNN feature used by IRS was trained on CUHK03.

***Evaluation on CUHK03*** The person re-id performance of different methods as compared to the IRS model on CUHK03 (Li et al. 2014) is reported in Table 4. We tested on both the manually labelled and automatically detected bounding boxes. Similar to VIPeR and CUHK01, our IRS model surpassed clearly all compared methods in either single- or multi-feature setting given manually labelled bounding boxes. Importantly, this advantage remains when more challenging detected bounding boxes were used, whilst other strong models such as NFST and GOG suffered more significant performance degradation. This shows both the robustness of our IRS model against misalignment and its greater scalability to real-world deployments.

***Evaluation on Market-1501*** We evaluated the re-id performance of existing models against the proposed IRS model on the Market-1501 benchmark (Zheng et al. 2015). The bounding boxes of all person images of this dataset were generated by an automatic pedestrian detector. Hence, this dataset presents a more realistic challenge to re-id models than conventional re-id datasets with manually labelled bounding boxes. Table 5 shows the clear superiority of our IRS model over all competitors. In particular, our IRS model achieved Rank-1 $73.9\%$ for single-query and Rank-1 $81.4\%$ for multi-query, significantly better than the strongest alternative method, the deep Gated S-CNN model (Varior et al. 2016a), by $8.1\%\,(73.9{-}65.8)$ (single-query) and $5.4\%\,(81.4{-}76.0)$ (multi-query). Similar advantages hold when compared using the mAP metric.

In summary, these comparative evaluations on the performance of batch-wise re-id model learning show that the IRS model outperforms comprehensively a wide range of existing re-id methods including both hand-crafted and deep learning based models. This validates the effectiveness and advantages of learning a re-id discriminative feature embedding using the proposed approach on identity regression.

**Table 3:** Re-id performance comparison on the CUHK01 benchmark

Dataset	CUHK01 (486/485 split)			
Rank (%)	R1	R5	R10	R20
kLFDA (Xiong et al. 2014)	54.6	80.5	86.9	92.0
XQDA (Liao et al. 2015)	63.2	83.9	90.0	94.2
MLAPG (Liao and Li 2015)	64.2	85.4	90.8	94.9
NFST (Zhang et al. 2016a)	65.0	85.0	89.9	94.4
HER (Wang et al. 2016b)	68.3	86.7	92.6	96.2
DCNN$+$ (Ahmed et al. 2015)	47.5	71.6	80.3	87.5
MCP (Cheng et al. 2016)	53.7	84.3	91.0	93.3
DGD (Xiao et al. 2016)	66.6	–	–	–
**IRS (WHOS)**	48.8	73.4	81.1	88.3
**IRS (LOMO)**	68.3	86.7	92.6	96.2
**IRS (CNN)**	**68.6**	**89.3**	**93.9**	**97.2**
ME$^{\mathrm{a}}$ (Paisitkriangkrai et al. 2015)	53.4	76.4	84.4	90.5
FFN-Net$^{\mathrm{a}}$ (Wu et al. 2016)	55.5	78.4	83.7	92.6
GOG$^{\mathrm{a}}$ (Matsukawa et al. 2016)	67.3	86.9	91.8	95.9
NFST$^{\mathrm{a}}$ (Zhang et al. 2016a)	69.1	86.9	91.8	95.4
HER$^{\mathrm{a}}$ (Wang et al. 2016b)	71.2	90.0	94.4	97.3
**IRS (WHOS + LOMO + CNN)** $^{\mathrm{a}}$	**80.8**	**94.6**	**96.9**	**98.7**

Dataset	CUHK01 (871/100 split)			
FPNN (Li et al. 2014)	27.9	59.6	73.5	87.3
DCNN$+$ (Ahmed et al. 2015)	65.0	–	–	–
JRL (Chen et al. 2016b)	70.9	92.3	96.9	98.7
EDM (Shi et al. 2016)	69.4	–	–	–
SICI (Wang et al. 2016a)	71.8	–	–	–
**IRS (WHOS)**	77.0	92.8	96.5	99.2
**IRS (LOMO)**	80.3	94.2	96.9	99.5
**IRS (CNN)**	84.4	98.2	**99.8**	**100**
**IRS (WHOS + LOMO + CNN)** $^{\mathrm{a}}$	**88.4**	**98.8**	99.6	**100**

Best results for single-feature and multi-feature are given in bold

$^{\mathrm{a}}$Multiple features fusion

**Table 4:** Re-id performance comparison on the CUHK03 benchmark

Dataset	CUHK03 (Manually)			
Rank (%)	R1	R5	R10	R20
kLFDA (Xiong et al. 2014)	45.8	77.1	86.8	93.1
XQDA (Liao et al. 2015)	52.2	82.2	92.1	96.3
MLAPG (Liao and Li 2015)	58.0	87.1	94.7	98.0
NFST (Zhang et al. 2016a)	58.9	85.6	92.5	96.3
HER (Wang et al. 2016b)	60.8	87.0	95.2	97.7
DCNN$+$ (Ahmed et al. 2015)	54.7	86.5	93.9	**98.1**
EDM (Shi et al. 2016)	61.3	–	–	–
DGD (Xiao et al. 2016)	75.3	–	–	
**IRS (WHOS)**	59.6	87.2	92.8	96.9
**IRS (LOMO)**	61.6	87.0	94.6	98.0
**IRS (CNN)**	**81.5**	**95.7**	**97.1**	98.0
ME$^{\mathrm{a}}$ (Paisitkriangkrai et al. 2015)	62.1	89.1	94.3	97.8
NFST$^{\mathrm{a}}$ (Zhang et al. 2016a)	62.6	90.1	94.8	98.1
HER$^{\mathrm{a}}$ (Wang et al. 2016b)	65.2	92.2	96.8	**99.1**
GOG$^{\mathrm{a}}$ (Matsukawa et al. 2016)	67.3	91.0	96.0	–
**IRS (WHOS + LOMO + CNN)** $^{\mathrm{a}}$	**81.9**	**96.5**	**98.2**	98.9

Dataset	CUHK03 (Detected)			
KISSME (Koestinger et al. 2012)	11.7	33.3	48.0	–
XQDA (Liao et al. 2015)	46.3	78.9	83.5	93.2
MLAPG (Liao and Li 2015)	51.2	83.6	92.1	96.9
L$_1$-Lap (Kodirov et al. 2016)	30.4	–	–	–
NFST (Zhang et al. 2016a)	53.7	83.1	93.0	94.8
DCNN$+$ (Ahmed et al. 2015)	44.9	76.0	83.5	93.2
EDM (Shi et al. 2016)	52.0	–	–	–
SICI (Wang et al. 2016a)	52.1	84.9	92.4	–
S-LSTM (Varior et al. 2016b)	57.3	80.1	88.3	–
Gated S-CNN (Varior et al. 2016a)	68.1	88.1	94.6	–
**IRS (WHOS)**	50.6	82.1	90.4	96.1
**IRS (LOMO)**	53.4	83.1	91.2	96.4
**IRS (CNN)**	**80.3**	**96.3**	**98.6**	**99.0**
NFST$^{\mathrm{a}}$ (Zhang et al. 2016a)	54.7	84.8	94.8	95.2
GOG$^{\mathrm{a}}$ (Matsukawa et al. 2016)	65.5	88.4	93.7	–
**IRS (WHOS + LOMO + CNN)** $^{\mathrm{a}}$	**83.3**	**96.2**	**97.9**	**98.6**

Best results for single-feature and multi-feature are given in bold

$^{\mathrm{a}}$Multiple features fusion

**(II) Effects of Embedding Space Design** To give more insight on why and how the IRS model works, we evaluated the effects of embedding space design in our IRS model. To this end, we compared the three coding methods as described in Sect. 3.2: *OneHot Feature Coding* in the proposed *Identity Regression Space*, *FDA Feature Coding* by Wang et al. (2016b), and *Random Feature Coding* by Zhu et al. (2016). In this experiment, we used the LOMO feature on all four datasets, the 485/486 people split on CUHK01, and the manually labelled bounding boxes on CUHK03. For random coding, we performed 10 times and used the averaged results to compare with the OneHot Feature Coding and the FDA Feature Coding. The results are presented in Table 6. We have the following observations:

(i) The embedding space choice plays a clear role in IRS re-id model learning and a more “semantic” aligned (both OneHot and FDA) coding has the advantage for learning a more discriminative IRS re-id model. One plausible reason is that the random coding may increase the model learning difficulty resulting in an inferior feature embedding, especially given the small sample size nature of re-id model learning. Instead, by explicitly assigning identity class “semantics” (prototypes) to individual dimensions of the embedding space, the feature embedding learning is made more selective and easier to optimise.
(ii) Both the OneHot and FDA Feature Coding methods yield the same re-id accuracy on both VIPeR and CUHK01. This is because on either dataset each training identity has the same number of images (2 for VIPeR and 4 for CUHK01), under which the FDA Coding (Eq. 4) is equivalent to the OneHot Feature Coding (Eq. 1).
(iii) Given the different image samples available per training person identity on CUHK03 and Market-1501, FDA Coding is slightly inferior to OneHot Feature Coding. This is interesting given the robust performance of FDA on conventional classification problems. Our explanation is rather straightforward if one considers the unique characteristics of the re-id problem where the training and test classes are *completely* non-overlapping. That is, the test classes have no training image samples. In essence, the re-id problem is conceptually similar to the problem of zero-shot learning (ZSL), in contrast to the conventional classification problems where test classes are sufficiently represented by the training data, i.e. totally overlapping. More specifically, learning by the FDA criterion optimises a model to the training identity classes given sufficient samples per class but it does not work well with small sample sizes, and more critically, it does *not necessarily* optimise the model for previously unseen test identity classes. This is because if the training identity population is relatively small, as in most re-id datasets, an unseen test person may not be similar to any of training people. That is, the distributions of the training and test population may differ significantly. Without any prior knowledge, a good representation of an unseen test class is some unique combination of all training persons *uniformly* without preference. Therefore, a feature embedding optimised uniformly without bias/weighting by the training class data sampling distribution is more likely to better cope with more diverse and unseen test classes, by better preserving class diversity in the training data *especially given the small sample size challenge* in re-id training data. This can be seen from the regularised properties of the OneHot Feature Coding in Sect. 3.

**(III) Effect of Features** We evaluated three different features (WHOS, LOMO, and CNN) individually and also their combinations used in our IRS model with the OneHot Feature Coding in Table 7. When a single type of feature is used, it is found that CNN feature is the best except on VIPeR, and LOMO is more discriminative than WHOS in most cases. The advantage of CNN feature over hand-crafted LOMO and WHOS is significant given larger training data in CUHK03 and Market-1501, yielding a *gain* of $19.9\%$ (CUHK03 (Manual)), $26.9\%$ (CUHK03 (Detected)), and $15.0\%$ (Market-1501) over LOMO in Rank-1. Without fine-tuning a CUHK03 trained model on the target domains, CNN feature still performs the best on CUHK01 due to the high similarity in view conditions between CUHK01 and CUHK03. CNN feature performs less well on VIPeR due to higher discrepancy in view conditions between VIPeR and CUHK03, i.e. the domain shift problem (Ma et al. 2013; Pan and Yang 2010).

We further evaluated multi-feature based performance by score-level fusion. It is evident that most combinations lead to improved re-id accuracy, and fusing all three features often generate the best results. This confirms the previous findings that different appearance information can be encoded by distinct features and their fusion enhances re-id matching (Paisitkriangkrai et al. 2015; Zhang et al. 2016a; Matsukawa et al. 2016; Chen et al. 2016a).

**Table 5:** Re-id performance comparison on the Market-1501 benchmark

Dataset	Market-1501			
Query per person	Single-query		Multi-query	
Metric (%)	R1	mAP	R1	mAP
KISSME (Koestinger et al. 2012)	40.5	19.0	–	–
MFA (Yan et al. 2007)	45.7	18.2	–	–
kLFDA (Xiong et al. 2014)	51.4	24.4	52.7	27.4
XQDA (Liao et al. 2015)	43.8	22.2	54.1	28.4
SCSP (Chen et al. 2016a)	51.9	26.3	–	–
NFST (Zhang et al. 2016a)	55.4	29.9	68.0	41.9
TMA (Martinel et al. 2016)	47.9	22.3	–	–
SSDAL (Su et al. 2016)	39.4	19.6	49.0	25.8
S-LSTM (Varior et al. 2016b)	–	–	61.6	35.3
Gated S-CNN (Varior et al. 2016a)	65.8	39.5	76.0	48.4
**IRS (WHOS)**	55.2	27.5	60.3	33.5
**IRS (LOMO)**	57.7	29.0	68.0	37.8
**IRS (CNN)**	**72.7**	**48.1**	**80.2**	**58.5**
SCSP$^{\mathrm{a}}$ (Chen et al. 2016a)	51.9	26.4	–	–
NFST$^{\mathrm{a}}$ (Zhang et al. 2016a)	61.0	35.7	71.6	46.0
**IRS (WHOS + LOMO + CNN)** $^{\mathrm{a}}$	**73.9**	**49.4**	**81.4**	**59.9**

Best results for single-feature and multi-feature are given in bold

$^{\mathrm{a}}$Multiple features fusion

**Table 6:** Effects of embedding space on re-id performance in our proposed IRS model

Dataset	VIPeR				CUHK01				CUHK03				Market-1501			
Rank (%)	R1	R5	R10	R20	R1	R5	R10	R20	R1	R5	R10	R20	R1 (SQ)	mAP (SQ)	R1(MQ)	mAP (MQ)
OneHot Feature Coding	**45.1**	**74.6**	**85.1**	**93.3**	**68.3**	**86.7**	**92.6**	**96.2**	**61.6**	**87.0**	94.6	**98.0**	**57.7**	**29.0**	**68.0**	**37.8**
FDA Feature Coding	**45.1**	**74.6**	**85.1**	**93.3**	**68.3**	**86.7**	**92.6**	**96.2**	60.8	**87.0**	**95.2**	97.7	55.6	27.5	67.5	36.8
Random Feature Coding	44.8	73.4	84.8	92.7	61.3	83.4	89.5	94.2	51.7	79.4	87.4	93.0	47.4	21.1	48.5	23.2

Best results for single-feature and multi-feature are given in bold

The LOMO visual feature were used on all datasets. We adopted the 485/486 people split on CUHK01 and the manually labelled person images on CUHK03

*SQ* single-query, *MQ* multi-query

**Table 7:** Effects of feature choice in re-id performance using the IRS model with OneHot Feature Coding

Dataset	VIPeR			
Rank (%)	R1	R5	R10	R20
WHOS (Lisanti et al. 2015)	44.5	**75.0**	**86.3**	**93.6**
LOMO (Liao et al. 2015)	**45.1**	74.6	85.1	93.3
CNN (Xiao et al. 2016)	33.1	59.9	71.5	82.2
WHOS + LOMO	53.0	79.8	89.6	95.5
CNN + LOMO	49.9	77.5	86.9	93.8
WHOS + CNN	49.7	78.0	87.9	94.4
WHOS + LOMO + CNN	**54.6**	**81.5**	**90.3**	**95.7**

Dataset	CUHK01 (486/485 split)			
WHOS (Lisanti et al. 2015)	48.8	73.4	81.1	88.3
LOMO (Liao et al. 2015)	68.3	86.7	92.6	96.2
CNN (Xiao et al. 2016)	**68.6**	**89.3**	**93.9**	**97.2**
WHOS + LOMO	71.2	90.0	94.4	97.3
CNN + LOMO	79.8	93.6	96.3	98.2
WHOS + CNN	76.1	92.9	96.1	98.2
WHOS + LOMO + CNN	**80.8**	**94.6**	**96.9**	**98.7**

Dataset	CUHK01 (871/100 split)			
WHOS (Lisanti et al. 2015)	77.0	92.8	96.5	99.2
LOMO (Liao et al. 2015)	80.3	94.2	96.9	99.5
CNN (Xiao et al. 2016)	**84.4**	**98.2**	**99.8**	**100**
WHOS + LOMO	83.6	95.4	98.8	**100**
CNN + LOMO	88.0	98.3	99.5	**100**
WHOS + CNN	**89.0**	98.5	**99.6**	**100**
WHOS + LOMO + CNN	88.4	**98.8**	**99.6**	**100**

Dataset	CUHK03 (Manually)			
WHOS (Lisanti et al. 2015)	59.6	87.2	92.8	96.9
LOMO (Liao et al. 2015)	61.6	87.0	94.6	98.0
CNN (Xiao et al. 2016)	**81.5**	**95.7**	**97.1**	**98.0**
WHOS + LOMO	65.2	92.2	96.8	**99.1**
CNN + LOMO	**82.6**	96.0	97.5	98.6
WHOS + CNN	80.4	95.7	98.0	98.4
WHOS + LOMO + CNN	81.9	**96.5**	**98.2**	98.9

Dataset	CUHK03 (Detected)			
WHOS (Lisanti et al. 2015)	50.6	82.1	90.4	96.1
LOMO (Liao et al. 2015)	53.4	83.1	91.2	96.4
CNN (Xiao et al. 2016)	**80.3**	**96.3**	**98.6**	**99.0**
WHOS + LOMO	59.9	89.4	95.5	98.5
CNN + LOMO	82.4	95.7	97.4	98.4
WHOS + CNN	81.1	95.4	97.5	**98.6**
WHOS + LOMO + CNN	**83.3**	**96.2**	**97.9**	**98.6**

Dataset	Market-1501			
Query Per Person	Single-Query		Multi-Query	
Metric (%)	R1	mAP	R1	mAP
WHOS (Lisanti et al. 2015)	55.2	27.5	60.3	33.5
LOMO (Liao et al. 2015)	57.7	29.0	68.0	37.8
CNN (Xiao et al. 2016)	**72.7**	**48.1**	**80.2**	**58.5**
WHOS + LOMO	62.4	33.6	69.0	41.0
CNN + LOMO	73.0	48.5	80.9	59.1
WHOS + CNN	72.8	48.3	80.3	58.7
WHOS + LOMO + CNN	**73.9**	**49.4**	**81.4**	**59.9**

Best results for single-feature and multi-feature are given in bold

**Table 8:** Evaluation on deep learning regression (DLR) on CUHK03 (Manually)

Rank (%)	R1	R5	R10	R20
CNN feature	73.7	91.5	95.0	97.2
Softmax prediction	73.3	91.0	93.9	96.4
**IRS** (DLR$^{1\text {-FC}}$)	75.1	92.7	95.3	97.5
**IRS** (DLR$^{2\text {-FC}}$)	76.6	93.1	95.9	**98.1**
**IRS** (DLR$^{3\text {-FC}}$)	74.2	92.5	94.8	97.1
CNN + **IRS** (RR)	**81.5**	**95.7**	**97.1**	98.0

Best results for single-feature and multi-feature are given in bold

Deep model: DGD (Xiao et al. 2016)

$\hbox {DLR}^{{n}\text {-FC}}$: $n\in \{1,2,3\}$ FC layers added in DLR

*RR* ridge regression

**(IV) Deep Learning Regression** Apart from the ridge regression (RR) algorithm (Hoerl and Kennard 1970; Zhang et al. 2010), the IRS concept can be also realised in deep learning, i.e. deep learning regression (DLR). We call this IRS implementation as **IRS (DLR)**. For this experiment, we adopted the DGD CNN model (Xiao et al. 2016) and the CUHK03 (Manual) dataset. In training IRS (DLR), we first trained the DGD to convergence with the softmax cross-entropy loss. Then, we added $n (=1,2,3)$ new 512-dim FC layers (including ReLU activation) with random parameter initialisation on top of DGD. Finally, we frozen all original DGD layers and optimised the new layers only by $L_2$ loss.

In this test, we compared with the DGD (1) CNN Features and (2) Softmax predictions (considered as some sort of IRS features although not strictly the same due to different modelling designs). We observed in Table 8 that: (1) IRS (DLR) outperforms both CNN Features and Softmax Prediction. This indicates the benefit of IRS in a deep learning context. (2) IRS (DLR) is relatively inferior to CNN + IRS (RR), suggesting that a deep learning model is not necessarily superior in regressing IRS when given limited training data. Moreover, IRS (RR) is superior on model learning efficiency, hence more suitable for incremental model update.

**Table 9:** Evaluation on the complementary effect of deep model pre-training based transfer learning (TL) and IRS on VIPeR

Rank (%)	R1	R5	R10	R20
$\hbox {W/O TL}^{\mathrm{a}}$	12.3	–	–	–
W TL	34.1	66.3	76.2	83.7
TL + **IRS** (RR)	**39.9**	**70.6**	**79.3**	**86.2**

Best results for single-feature and multi-feature are given in bold

Deep model: DGD (Xiao et al. 2016)

$^{\mathrm{a}}$Reported result in Xiao et al. (2016)

**(V) Complementary of Transfer Learning and IRS** Transfer learning (TL) is another independent scheme for solving the SSS problem. We tested the benefit of deep learning pre-trained TL and IRS. We evaluated three methods based on the DGD Xiao et al. (2016): (1) ***W/O TL***: Trained the DGD on VIPeR training data (632 images) only. (2) ***W TL***: First pre-trained the DGD on 26,246 CUHK03 images for knowledge transfer learning, then fine-tuned on the VIPeR training data. (3) ***TL + IRS (RR)***: First adopted the CUHK03 pre-trained and VIPeR fine-tuned DGD to extract CNN features, then deployed the ridge regression based IRS to train the final re-id feature embedding model. All three models were evaluated on the same VIPeR test data. Table 9 shows that: (1) Pre-training based TL significantly improves re-id performance. This demonstrates the benefit of TL in solving the SSS problem. (2) IRS clearly further improves the re-id accuracy. This verifies the additional benefits of IRS and the complementary advantage of TL and IRS to a deep learning model for solving the SSS challenge.

**Table 10:** Comparing subspace learning models with different features

Dataset–feature	VIPeR–WHOS			
Rank (%)	R1	R5	R10	R20
KISSME (Koestinger et al. 2012)	28.7	57.2	72.6	86.1
kLFDA (Xiong et al. 2014)	40.1	68.5	81.2	91.7
XQDA (Liao et al. 2015)	35.1	63.9	74.9	86.0
NFST (Zhang et al. 2016a)	43.6	74.1	86.1	92.7
IRS	**44.5**	**75.0**	**86.3**	**93.6**

Dataset–feature	VIPeR–LOMO			
KISSME (Koestinger et al. 2012)	22.1	53.4	68.8	83.8
kLFDA (Xiong et al. 2014)	38.6	69.2	80.4	89.2
XQDA (Liao et al. 2015)	40.0	68.1	80.5	91.1
NFST (Zhang et al. 2016a)	42.3	71.5	82.9	92.1
IRS	**45.1**	**74.6**	**85.1**	**93.3**

Dataset–feature	VIPeR–CNN			
KISSME (Koestinger et al. 2012)	22.6	46.9	59.0	72.7
kLFDA (Xiong et al. 2014)	30.9	55.6	65.7	75.0
XQDA (Liao et al. 2015)	11.7	26.2	35.5	48.1
NFST (Zhang et al. 2016a)	31.2	56.0	67.2	78.4
IRS	**33.1**	**59.9**	**71.5**	**82.2**

Dataset–feature	CUHK03(M)–WHOS			
Rank (%)	R1	R5	R10	R20
KISSME (Koestinger et al. 2012)	31.6	63.4	76.6	88.3
kLFDA (Xiong et al. 2014)	32.9	59.2	75.7	82.6
XQDA (Liao et al. 2015)	41.1	66.5	77.2	86.6
NFST (Zhang et al. 2016a)	34.4	59.7	68.2	77.6
IRS	**59.6**	**87.2**	**92.8**	**96.9**

Dataset–feature	CUHK03(M)–LOMO			
KISSME (Koestinger et al. 2012)	32.7	68.0	81.3	91.4
kLFDA (Xiong et al. 2014)	45.8	77.1	86.8	93.1
XQDA (Liao et al. 2015)	52.2	82.2	92.1	96.3
NFST (Zhang et al. 2016a)	58.9	85.6	92.5	96.3
IRS	**61.6**	**87.0**	**94.6**	**98.0**

Dataset–feature	CUHK03(M)–CNN			
KISSME (Koestinger et al. 2012)	73.8	94.0	96.2	**98.0**
kLFDA (Xiong et al. 2014)	76.0	92.3	96.0	**98.0**
XQDA (Liao et al. 2015)	70.8	92.0	96.2	97.9
NFST (Zhang et al. 2016a)	62.6	78.9	85.5	89.7
IRS	**81.5**	**95.7**	**97.1**	**98.0**

Best results for single-feature and multi-feature are given in bold

**(VI) Comparisons to Subspace/Metric Learning Models** We performed comparative experiments on four subspace and metric learning models including KISSME (Koestinger et al. 2012), kLFDA (Xiong et al. 2014), XQDA (Liao et al. 2015), and NFST (Zhang et al. 2016a), using three different types of features (WHOS, LOMO, CNN) and identical training/test data. We utilised the same subspace dimension for XQDA and our IRS, i.e. the number of training person classes. We conducted this evaluation on VIReR and CUHK03 (Manual). Table 10 shows that the proposed IRS model consistently surpasses all the compared alternative models. This again suggests the advantages of IRS in learning discriminative re-id models.

**Fig. 7:**
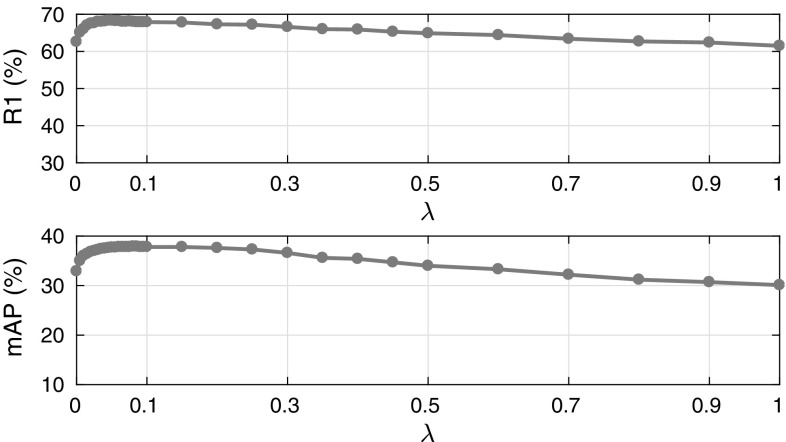
Regularisation sensitivity on the Market-1501 dataset. The multi-query setting was used

**(VII) Regularisation Sensitivity** We analysed the sensitivity of the only free parameter $\lambda$ in Eq. (3) which controls the regularisation strength of our IRS model. This evaluation was conducted with the LOMO feature in the multi-query setting on Market-1501 (Zheng et al. 2015). Specifically, we evaluated the Rank-1 and mAP performance with $\lambda$ varying from 0 to 1. Figure 7 shows that our IRS model has a large satisfactory range of $\lambda$ and therefore not sensitive. We set $\lambda = 0.1$ in all evaluations.

**Table 11:** Model complexity and training costs of person re-id models

Dataset	VIPeR	CUHK01	CUHK03	Market-1501
Training size	632	1940	12,197	12,936
MLAPG	50.9	746.6	$4.0\times 10^4$	–
kLFDA	5.0	45.9	2203.2	1465.8
XQDA	4.1	51.9	3416.0	3233.8
NFST	1.3	6.0	1135.1	801.8
**IRS**	**1**.**2**	**4**.**2**	**248.8**	**266.3**

Best results for single-feature and multi-feature are given in bold

*Metric*: Model training time (in s), smaller is better

**(VIII) Model Complexity** In addition to model re-id accuracy, we also examined the model complexity and computational costs, in particular model training time. We carried out this evaluation by comparing our IRS model with some strong metric learning methods including kLFDA (Xiong et al. 2014), XQDA (Liao et al. 2015), MLAPG (Liao and Li 2015), and NFST (Zhang et al. 2016a). Given *n* training samples represented by *d*-dimensional feature vectors, it requires $\frac{3}{2}dnm\,+\,\frac{9}{2}m^3$ ($m = \min (d,n)$) floating point addition and multiplications (Penrose 1955) to perform an eigen-decomposition for solving either a generalised eigen-problem (Xiong et al. 2014; Liao et al. 2015) or a null space (Zhang et al. 2016a), whereas solving the linear system of the IRS model (Eq. 3) takes $\frac{1}{2}dnm\,+\,\frac{1}{6}m^3$ (Cai et al. 2008). Deep learning models (Ahmed et al. 2015; Xiao et al. 2016; Varior et al. 2016a) are not explicitly evaluated since they are usually much more demanding in computational overhead, requiring much more training time (days or even weeks) and more powerful hardware (GPU). In this evaluation, we adopted the LOMO feature for all datasets and all the models compared, the 485/486 people split on CUHK01, the manually labelled person bounding boxes on CUHK03, and the single-query setting on Market-1501.

For each model, we recorded and compared the average training time of 10 trials performed on a workstation with 2.6 GHz CPU. Table 11 presents the training time of different models (in s). On the smaller VIPeR dataset, our IRS model training needed only 1.2 s, similar as NFST and 42.4 times faster than MLAPG. On larger datasets CUHK01, CUHK03 and Market-1501, all models took longer time to train and training the IRS model remains the fastest with speed-up over MLAPG enlarged to 177.8/160.8 times on CUHK01/CUHK03, respectively.^2^ This demonstrates the advantage of the proposed IRS model over existing competitors for scaling up to large sized training data.

### Incremental Person Re-id Evaluation

We further evaluated the performance of our IRS model using the incremental learning IRS$^\text {inc}$ algorithm (Sect. 4). This setting starts with a small number, e.g. 10 of labelled true match training pairs, rather than assuming a large pre-collected training set. Often, no large sized labelled data is available in typical deployments at varying scenes in advance. More labelled data will arrive one by one over time during deployment due to human-in-the-loop verification. In such a setting, a re-id model can naturally evolve through deployment life-cycle and efficiently adapt to each application test domain. In this context, we consider two incremental re-id model learning scenarios: **(I)** *Passive* incremental learning where unlabelled person images are randomly selected for human to verify; **(II)** *Active* incremental learning where person images are actively determined by the proposed JointE$^2$ active learning algorithm (Sect. 4.1).

**Incremental Re-id Evaluation Protocol** Due to the lack of access to large sized training samples in batch, incrementally learned models are typically less powerful than batch learned models (Poggio and Cauwenberghs 2001; Ristin et al. 2014). Therefore, it is critical to evaluate how much performance drop is introduced by the incremental learning (IL) algorithm, IRS$^\text {inc}$, as compared to the corresponding batch-wise learning (BL) and how much efficiency is gained by IL.

We started with 10 labelled identities, i.e. cross-camera truth matches of 10 persons, and set the total labelling budget to 200 persons. For simplicity, we selected four test cases with 50, 100, 150, 200 labelled identities respectively and evaluated their model accuracy and training cost. To compare the accumulated learning time (ALT),^3^ i.e. the summed time for training all the IRS models when the label number is increased from 50 to 200 one by one (in total 151 updates), we interpolated estimations on training time between these four measured test cases. A one-by-one model update is necessary particularly when deploying a pre-trained sub-optimal re-id model to a previously unseen camera network with weak starting performance.

We adopted the LOMO visual feature on all datasets. We utilised the 485/486 people split on CUHK01, the manually labelled person images on CUHK03, the single-query setting on Market-1501, and the same test data as the experiments in Sect. 5.1. We conducted 10 folds of evaluations each with a different set of random unlabelled identities and reported the averaged results.

**Table 12:** Comparing passive incremental learning (IL) versus batch-wise Learning (BL) using the IRS model

Dataset		VIPeR					CUHK01					CUHK03					Market-1501				
Label no.		50	100	150	200	*ALT*	50	100	150	200	*ALT*	50	100	150	200	*ALT*	50	100	150	200	*ALT*
Time (s)	BL	0.23	0.23	0.25	0.26	36.5	1.43	1.51	1.57	1.66	232.8	20.4	21.7	22.4	24.5	3349.9	119.5	121.5	125.6	140.3	$1.9 \times 10^4$
	IL	**0**.**02**	**0**.**02**	**0**.**02**	**0**.**03**	**3**.**28**	**0**.**14**	**0**.**15**	**0**.**16**	**0**.**17**	**23**.**4**	**1**.**62**	**1**.**69**	**1**.**70**	**1**.**81**	**257**.**0**	**1**.**94**	**5**.**05**	**6**.**61**	**9**.**60**	**877**.**3**
R1 (%)	BL	**20**.**6**	**29**.**2**	**34**.**9**	**38**.**9**	–	**21**.**9**	**37**.**3**	**46**.**5**	**52**.**5**	–	**24**.**0**	**35**.**2**	**40**.**5**	**43**.**8**	–	**28**.**6**	**44**.**5**	**51**.**7**	**55**.**2**	–
	IL	19.4	**29**.**2**	33.6	37.2	–	20.8	35.6	45.3	51.5	–	22.1	33.0	38.8	41.7	–	27.5	44.2	50.6	54.3	–

Best results for single-feature and multi-feature are given in bold

*ALT* accumulated learning time, i.e. the summed time for training all the 151 IRS models when the label number is increased from 50 to 200 one by one

**(I) Passive Incremental Learning** We compared the proposed incremental learning (IL) based IRS (IRS$^\text {inc}$) with the batch-wise learning (BL) based IRS in Table 12 for model training time and re-id Rank-1 performance. It is found that IRS model training speed can increase by one order of magnitude or more, with higher speed-up observed on larger datasets and resulting in more model training efficiency gain. Specifically, on VIPeR, BL took approximately 36.5 s to conduct the 151 model updates by re-training, while IL only required 3.28 s. When evaluated on Market-1501, BL took over 5.5 h ($1.9\times 10^4$ s) to perform the sequential model updates, while IL was more than $20\times$ faster, only took 877.3 s. Importantly, this speed-up is at the cost of only $1{-}2$% Rank-1 drop. This suggests an attractive trade-off for the IRS$^\text {inc}$ algorithm between effectiveness and efficiency in incremental model learning.

**(II) Active Incremental Learning** We further evaluated the effect of the proposed *Joint*$\text {E}^2$ active learning algorithm (Sect. 4.1) by random passive unlabelled image selection (*Random*). Also, we compared with a state-of-the-art density based active sampling method (Ebert et al. 2012) which prefers to query the densest region of unlabelled sample space (*Density*). For both active sampling methods, we used our IRS$^\text {inc}$ for re-id model training. We evaluated the four test cases (50, 100, 150, 200 labelled identities) as shown in Table 13.

It is evident from Table 13 that: (**1**) On all four datasets, our Joint$\text {E}^2$ outperformed clearly both *Random* and *Density* given varying numbers of labelled samples. For example, when 50 identities were labelled, the proposed Joint$\text {E}^2$ algorithm beats *Random* sampling in Rank-1 by $4.0\%\,(23.4{-} 19.4)$, $9.1\%\,(29.9{-}20.8)$, $3.0\%\,(25.1{-}22.1), 9.0\%\,(36.5{-} 27.5$) on VIPeR, CUHK01, CUHK03 and Market-1501, respectively. (**2**) Our Joint$\text {E}^2$ model obtained similar or even better performance with less human labelling effort. For example, on Market-1501, by labelling 150 identities, Joint$\text {E}^2$ achieved Rank-1 rate of $54.8\%$, surpassed random ($54.3\%$) and density ($53.9\%$) with a greater budget of 200 identities.

In summary, the results in Tables 12 and 13 show clearly that the hybrid of our proposed IRS$^\text {inc}$ model and Joint$\text {E}^2$ active sampling method provides a highly scalable active incremental re-id model training framework, with attractive model learning capability and efficiency from less labelling effort suited for real-world person re-id applications.

**Table 13:** Evaluation on the active incremental learning algorithm

Dataset	VIPeR				CUHK01				CUHK03				Market-1501			
Label no.	50	100	150	200	50	100	150	200	50	100	150	200	50	100	150	200
Random	19.4	29.2	33.6	37.2	20.8	35.6	45.3	51.5	22.1	33.0	38.8	41.7	27.5	44.2	50.6	54.3
Density (Ebert et al. 2012)	18.4	26.8	33.5	37.5	23.3	37.0	44.5	50.0	23.7	34.8	40.2	42.7	32.3	46.2	51.5	53.9
**Joint** $\mathbf {E}^2$	**23.4**	**31.4**	**36.5**	**40.9**	**29.9**	**39.7**	**47.1**	**52.2**	**25.1**	**36.8**	**41.3**	**43.0**	**36.5**	**50.7**	**54.8**	**58.2**

Best results for single-feature and multi-feature are given in bold

*Metric*: Rank-1 rate (%)

## Conclusion

In this work, we developed a novel approach to explicitly designing a feature embedding space for supervised batch-wise and incremental person re-identification model optimisation. We solved the re-id model learning problem by introducing an identity regression method in an identity regression space (IRS) with an efficient closed-form solution. Furthermore, we formulated an incremental learning algorithm IRS$^\text {inc}$ to explore sequential on-line labelling and model updating. This enables the model to not only update efficiently the re-id model once new data annotations become available, but also allows probably early re-id deployment and improves adaptively the re-id model to new test domains with potential temporal dynamics. To better leverage human annotation effort, we further derived a novel active learning method JointE$^2$ to selectively query the most informative unlabelled data on-line. Extensive experiments on four benchmarks show that our IRS method outperforms existing state-of-the-art re-id methods in the conventional batch-wise model learning setting. Moreover, the proposed incremental learning algorithm increases significantly model training speed, over 10 times faster than batch-wise model learning, by only sacrificing marginal model re-id capability with $1{-}2\%$ Rank-1 drop. This labelling-while-deploying strategy has the intrinsic potential of helping reduce the cost of manual labelling in large scale deployments by structuring semantically the unlabelled data so to expedite the true match identification process. Additionally, our active learning method improves notably the human labelling quality w.r.t. the thus-far model, particularly when limited budget is accessible, providing over $3\%$ Rank-1 improvement than Random sampling given 50 identities labelling budget. While person re-id has attracted increasing amount of efforts especially in the deep learning paradigm, model learning scalability, model incremental adaptation, and labelling effort minimisation in large scale deployments however are significantly underestimated although very critical in real-world applications. By presenting timely an effective solution in this work, we hope that more investigations towards these important problems will be made in the future studies. One interesting future direction is to develop incremental deep re-id learning algorithms.

## Appendix: Derivation of FDA Coding

In the following, we provide a detailed derivation of FDA coding (Eq. 4) in our IRS method.

*FDA Criterion* Specifically, the FDA criterion aims to minimise the intra-class (person) appearance variance and maximise inter-class appearance variance. Formally, given zero-centred training data $\varvec{X} = \{\varvec{x}_i\}_{i=1}^n$, we generate three scatter matrices defined as follows:

19 $$\begin{aligned} \begin{aligned} \varvec{S}_w&= \frac{1}{n}\sum _{j=1}^c \sum _{l_i=j} (\varvec{x}_i - \varvec{u}_j)(\varvec{x}_i - \varvec{u}_j)^\top , \\ \varvec{S}_b&= \frac{1}{n}\sum _{j=1}^c n_j \varvec{u}_j \varvec{u}_j^\top ,\\ \varvec{S}_t&= \varvec{S}_w\,+\,\varvec{S}_b= \frac{1}{n}\sum _{i=1}^n \varvec{x}_i \varvec{x}_i^\top , \end{aligned} \end{aligned}$$

where $\varvec{S}_w, \varvec{S}_b$, and $\varvec{S}_t$ denote *within-class*, *between-class* and *total* scatter matrices respectively, $\varvec{u}_j$ the class-wise centroids, and $n_j$ the sample size of the *j*th class (or person). The objective function of FDA aims at maximising ${ trace}(\varvec{S}_b)$ and minimising ${ trace} (\varvec{S}_w)$ simultaneously, where $\varvec{S}_w$ can be replaced by $\varvec{S}_t$ since $\varvec{S}_t = \varvec{S}_b\,+\,\varvec{S}_w$. Hence, an optimal transformation $\varvec{G}^*$ by FDA can be computed by solving the following problem:

20 $$\begin{aligned} \varvec{G}^* = \arg \max _{\varvec{G}} \; {{ trace}} \Big (\big (\varvec{G}^\top \varvec{S}_b\varvec{G}\big )\big (\varvec{G}^\top \varvec{S}_t\varvec{G}\big )^\dagger \Big ). \end{aligned}$$

### Theorem 1

With $\varvec{Y}$ defined as Eq. (4), the projection $\varvec{P}^*$ learned by Eq. (3) is equivalent to $\varvec{G}^*$, the optimal FDA solution in Eq. (20).

### Proof

First, optimising the objective in Eq. (4) involves solving the following eigen-problem:

21 $$\begin{aligned} \varvec{S}_t^\dagger \varvec{S}_b\varvec{G} = \varvec{G}\varvec{\varLambda }, \end{aligned}$$

where $\varvec{G} \in {\mathbb {R}}^{d\times q} = \left[ \varvec{g}_1,\ldots , \varvec{g}_q\right]$ contains *q* eigenvectors of $\varvec{S}_t^\dagger \varvec{S}_b$, and $\varvec{\varLambda } = { diag}(\alpha _1, \ldots , \alpha _q)$ with $\alpha _i$ the corresponding eigenvalue, and $q = { rank}(\varvec{S}_b) \le c - 1$. From the definitions in Eqs. (4) and (19), $\varvec{S}_t$ and $\varvec{S}_b$ can be further expanded as:

22 $$\begin{aligned} \varvec{S}_t&= \varvec{X}\varvec{X}^\top , \quad \varvec{S}_b = \varvec{X}\varvec{Y}\varvec{Y}^\top \varvec{X}^\top . \end{aligned}$$

Here, the multiplier $\frac{1}{n}$ is omitted in both scatter matrices for simplicity. Now, we can rewrite the left-hand side of Eq. (21) as:

23 $$\begin{aligned} (\varvec{X}\varvec{X}^\top \,+\,\lambda \varvec{I})^\dagger \varvec{X}\varvec{Y}\varvec{Y}^\top \varvec{X}^\top \varvec{G} = \varvec{G}\varvec{\varLambda }. \end{aligned}$$

Note that, the pseudo-inverse $\varvec{S}_t^\dagger$ is calculated by $(\varvec{X}\varvec{X}^\top \,+\,\lambda \varvec{I})^\dagger$. The reason is that in real-world problems such as person re-id where training data is often less sufficient, $\varvec{S}_t$ is likely to be ill-conditioned, i.e. singular or close to singular, so that its inverse cannot be accurately computed.

By our solution $\varvec{P}$ in Eq. (3), we can further rewrite Eq. (23):

24 $$\begin{aligned} \varvec{P}\varvec{Y}^\top \varvec{X}^\top \varvec{G} = \varvec{G}\varvec{\varLambda } \end{aligned}$$

To connect the regression solution $\varvec{P}$ and the FDA solution $\varvec{G}$, we define a $c\times c$ matrix $\varvec{R} = \varvec{Y}^\top \varvec{X}^\top \varvec{P}$. According to the general property of eigenvalues (Horn and Johnson 2012), $\varvec{R}$ and $\varvec{P}\varvec{Y}^\top \varvec{X}^\top$ share the same *q* non-zero eigenvalues. Also, if $\varvec{V} \in {\mathbb {R}}^{c\times q}$ contains the *q* eigenvectors of $\varvec{R}$, columns of the matrix $\varvec{P}\varvec{V}$ must be the eigenvectors of the matrix $\varvec{P}\varvec{Y}^\top \varvec{X}^\top$. Therefore, the relation between $\varvec{P}$ and $\varvec{G}$ is:

25 $$\begin{aligned} \varvec{G} = \varvec{P}\varvec{V} \end{aligned}$$

Finally, we show in the following Lemma that $\varvec{P}$ and $\varvec{G}$ are equivalent in the aspect of re-id matching. $\square$

### Lemma 1

In the embedding provided by $\varvec{P}$ and $\varvec{G}$, the nearest neighbour algorithm produce same result. That is, $(\varvec{x}_i - \varvec{x}_j)^\top \varvec{P}\varvec{P}^\top (\varvec{x}_i - \varvec{x}_j) = (\varvec{x}_i - \varvec{x}_j)^\top \varvec{G}\varvec{G}^\top (\varvec{x}_i - \varvec{x}_j)$.

### Proof

The necessary and sufficient condition for Lemma 1 is $\varvec{P}\varvec{P}^\top = \varvec{G}\varvec{G}^\top$. As $\varvec{V} \in {\mathbb {R}}^{c\times q}$, there must exist a matrix $\varvec{V}_2 \in {\mathbb {R}}^{c\times (c-q)}$ such that $\varvec{\hat{V}} = [\varvec{V}, \varvec{V}_2]$ is a $c\times c$ orthogonal matrix. Suppose the diagonal matrix $\varvec{\varGamma }$ contains the non-zero eigenvalues of $\varvec{R}$, then the eigen decomposition $\varvec{R}=\varvec{V}\varvec{\varGamma }\varvec{V}^\top$ implies that $\varvec{V}_2^\top \varvec{R}\varvec{V}_2 = 0$. $\square$

Recall that $\varvec{R} = \varvec{Y}^\top \varvec{X}^\top \varvec{P}$, and $\varvec{P} = (\varvec{X}\varvec{X}^\top \,+\,\lambda \varvec{I})^\dagger \varvec{X}\varvec{Y}$, then we obtain:

26 $$\begin{aligned} \varvec{V}_2^\top \varvec{Y}^\top \varvec{X}^\top (\varvec{X}\varvec{X}^\top \,+\,\lambda \varvec{I})^\dagger \varvec{X}\varvec{Y} \varvec{V}_2 = 0 \end{aligned}$$

As $(\varvec{X}\varvec{X}^\top \,+\,\lambda \varvec{I})^\dagger$ is positive definite, the above equation implies that $\varvec{X}\varvec{Y}\varvec{V}_2 = 0$, and hence $\varvec{P}\varvec{V}_2 =(\varvec{X}\varvec{X}^\top \,+\,\lambda \varvec{I})^\dagger \varvec{X}\varvec{Y} \varvec{V}_2 = 0$. Hence, we have:

27 $$\begin{aligned} \begin{aligned} \varvec{P}\varvec{P}^\top&= \varvec{P}\varvec{\hat{V}}\varvec{\hat{V}}^\top \varvec{P}^\top \\&= \varvec{P}\varvec{V}\varvec{V}^\top \varvec{P}^\top \,+\,\varvec{P}\varvec{V}_2\varvec{V}_2^\top \varvec{P}^\top \\&= \varvec{G}\varvec{G}^\top \,+\,0 \end{aligned} \end{aligned}$$

As such, the proof to Lemma 1 and Theorem 1 is complete.

## Footnotes

1 The HER model presented in our preliminary work (Wang et al. 2016b) is the same as IRS (LOMO) with FDA coding (Eq. 4), i.e. HER = IRS-FDA (LOMO). On the other hand, IRS (LOMO) in Tables 2, 3, 4 and 5 is IRS-OneHot (LOMO). The effects of choosing different coding is evaluated later (Table 6).

2 The MLAPG model failed to converge on Market-1501.

3 The BL model needs to be trained once only after all 200 person classes are labelled when we consider the batch-wise model learning setting (Sect. 5.1). However, here we consider instead the incremental learning setting with the aim to evaluate the proposed incremental learning algorithm in both training efficiency and effectiveness, as compared to the batch learning counterpart when deployed for model incremental update. Given the batch-wise learning strategy, incremental model update can only be achieved by re-training a model from scratch. Therefore, the accumulated learning time is a rational metric for efficiency comparison in this context.
